# The Reaction Specificity of Mammalian ALOX15 Orthologs is Changed During Late Primate Evolution and These Alterations Might Offer Evolutionary Advantages for Hominidae

**DOI:** 10.3389/fcell.2022.871585

**Published:** 2022-04-21

**Authors:** Dagmar Heydeck, Florian Reisch, Marjann Schäfer, Kumar R. Kakularam, Sophie A. Roigas, Sabine Stehling, Gerhard P. Püschel, Hartmut Kuhn

**Affiliations:** ^1^ Department of Biochemistry, Charité—Universitätsmedizin Berlin, Corporate Member of Freie Universität Berlin and Humboldt Universität zu Berlin, Berlin, Germany; ^2^ Institute for Nutritional Sciences, University Potsdam, Potsdam, Germany

**Keywords:** eicosanoids, lipid peroxidation, oxidative stress, recombinant proteins, ferroptosis

## Abstract

Arachidonic acid lipoxygenases (ALOXs) have been implicated in the immune response of mammals. The reaction specificity of these enzymes is decisive for their biological functions and ALOX classification is based on this enzyme property. Comparing the amino acid sequences and the functional properties of selected mammalian ALOX15 orthologs we previously hypothesized that the reaction specificity of these enzymes can be predicted based on their amino acid sequences (Triad Concept) and that mammals, which are ranked in evolution below gibbons, express arachidonic acid 12-lipoxygenating ALOX15 orthologs. In contrast, Hominidae involving the great apes and humans possess 15-lipoxygenating enzymes (Evolutionary Hypothesis). These two hypotheses were based on sequence data of some 60 mammalian ALOX15 orthologs and about half of them were functionally characterized. Here, we compared the ALOX15 sequences of 152 mammals representing all major mammalian subclades expressed 44 novel ALOX15 orthologs and performed extensive mutagenesis studies of their triad determinants. We found that *ALOX15* genes are absent in extant *Prototheria* but that corresponding enzymes frequently occur in *Metatheria* and *Eutheria*. More than 90% of them catalyze arachidonic acid 12-lipoxygenation and the Triad Concept is applicable to all of them. Mammals ranked in evolution above gibbons express arachidonic acid 15-lipoxygenating ALOX15 orthologs but enzymes with similar specificity are only present in less than 5% of mammals ranked below gibbons. This data suggests that ALOX15 orthologs have been introduced during *Prototheria*-*Metatheria* transition and put the Triad Concept and the Evolutionary Hypothesis on a much broader and more reliable experimental basis.

## Introduction

Arachidonic acid lipoxygenases (ALOX-isoforms) are lipid-peroxidizing enzymes converting polyunsaturated fatty acids (PUFAs) to the corresponding hydroperoxy derivatives (Haeggström and Funk, 2011; Kuhn et al., 2015; Mashima and Okuyama, 2015). The human genome involves six functional *ALOX* genes, a number of functionless pseudogenes and an *ALOX12* antisense gene with unknown functionality (Kuhn et al., 2018). Most of these genes have been mapped to a joint *ALOX* gene cluster localized on the short arm of chromosome 17 (Funk et al., 2002), but the *ALOX5* gene, which encodes for the key enzyme of leukotriene biosynthesis (Rådmark et al., 2015)**,** is located on chromosome 10 (Funk et al., 2002). With arachidonic acid (AA) as substrate human ALOX paralogs exhibit different reaction specificities and AA 15-lipoxygenating [ALOX15 (Bryant et al., 1982), ALOX15B (Brash et al., 1997)], AA 12-lipoxygenating [ALOX12 (Hamberg and Samuelsson, 1974), ALOX12B (Boeglin et al., 1998)] and AA 5-lipoxygenating [human ALOX5 (Rådmark et al., 2015)] enzymes have been described. Under physiological conditions ALOXE3 does not exhibit a fatty acid oxygenase activity but functions as lipid hydroperoxide isomerase (Zheng and Brash, 2010). In mice, the situation is somewhat different. The mouse genome involves a single ortholog for each human *ALOX* gene and as in humans, most *Alox* genes are localized in a joint gene cluster present in a syntenic region on mouse chromosome 11. In this gene cluster a functional *Aloxe12* gene was detected, which is a corrupted pseudogene in humans (Kuhn et al., 2018). The biological function of Aloxe12 is still unknown but multiple amino acid alignments revealed a high degree of evolutionary relatedness with mouse and human ALOX15 (Kuhn et al., 2018).

**SCHEME 1:**
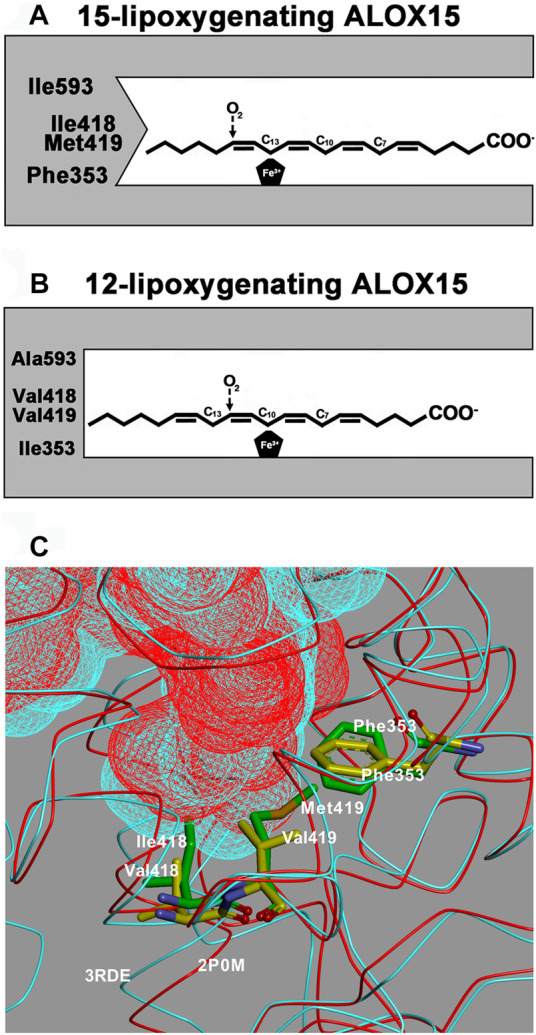
The Triad Concept of reaction specificity of mammalian ALOX15 orthologs. A + B) Schematic view of the Triad Concept: Arachidonic acid is aligned at the active site of ALOX15 orthologs in such a way that the methyl tail of the fatty acid substrate penetrates into the substrate binding pocket. The bisallylic methylenes C_13_ [for AA 15-lipoxygenating ALOX15 orthologs, **(A)**] or C_10_ [for AA 12-lipoxygenating ALOX15 orthologs, **(B)**] are localized in close proximity to the enzyme bound iron, which allows hydrogen abstraction from these carbon atoms. Molecular dioxygen is subsequently introduced at C_15_ (for AA 15-lipoxygenating ALOX15 orthologs) or at C_12_ (for AA 12-lipoxygenating ALOX15 orthologs). **(C)** Comparison of crystal structures: The substrate binding cavities of rabbit ALOX15 (PDB entry number 2P0M, red) and pig ALOX15 (PDB entry number 3RDE, turquoise) are overlaid and the major triad determinants [Phe(F)353, Ile(I)418 + Met(M)419 for rabbit ALOX15 and Phe(F)353, Val(V)418 + Val(V)419 for pig ALOX15] are indicated. It can be seen that the substrate binding pocket for the pig enzyme is deeper and this allows a more comprehensive penetration of the substrate fatty acid into its binding pocket. Thus, arachidonic acid is aligned at the active site in a way that favors 12-lipoxygenation.

Among mammalian ALOX isoforms, the ALOX15 orthologs are somewhat peculiar because of two catalytic properties: 1) these enzymes are capable of oxygenating PUFAs, which are bound to ester lipids of biomembranes (Schewe et al., 1975; Kuhn et al., 1990) and lipoproteins (Belkner et al., 1998; Kühn et al., 1994). Because of this catalytic activity, the corresponding enzymes have been implicated in targeted organelle degradation during cell differentiation (Rapoport and Schewe, 1986; van Leyen et al., 1998) but also in the pathogenesis of atherosclerosis (Cyrus et al., 1999; Kühn et al., 1997). 2) Mammalian ALOX15 orthologs exhibit variable reaction specificities. The human enzyme functions as AA 15-lipoxygenating ALOX15 (Sigal et al., 1990; Kühn et al., 1993) converting this PUFA to the corresponding 15-hydroperoxy derivative (15S-HpETE). On the other hand, mouse (Freire-Moar et al., 1995; Chen et al., 1994), rat (Watanabe and Haeggstrom, 1993; Pekárová et al., 2015), pig (Yokoyama et al., 1986) and cow (De Marzo et al., 1992) ALOX15 orthologs catalyze AA 12-lipoxygenation. A structural model explaining the variable reaction specificities of different mammalian ALOX15 orthologs has been introduced (Sloane et al., 1991; Borngräber et al., 1996; Suzuki et al., 1994; Sloane et al., 1995) and on the basis of this data the Triad Concept was worked out (Borngräber et al., 1999; Ivanov et al., 2010). This concept suggested that three clusters of amino acids [Phe(F)353, Borngraber-1 determinant; Ile(I)418 + Met(M)419, Sloane determinants; Ile(I)593 + Thr(T)594, Borngraber-2 determinants; amino acid numbering for human ALOX15] form the bottom of the substrate binding pocket and that the side chain geometry of these residues determines the depth of this cavity (Borngräber et al., 1999; Ivanov et al., 2010). If small amino acids are localized at these positions, the substrate fatty acids may penetrate deeply into the pocket, which allows AA 12-lipoxygenation. In contrast, when more space-filling residues are located at these positions, AA 15-lipoxygenation is favored (Scheme 1; Supplementary Video S1). When the less space-filling triad determinants of AA 12-lipoxygenating ALOX15 orthologs were mutated to more bulky residues AA 15-lipoxygenation was induced (Borngräber et al., 1999; Ivanov et al., 2010).

During the early days of ALOX15 research, only limited structural information on mammalian ALOX15 orthologs was available and thus, it was impossible to explore whether the variable reaction specificities of mammalian ALOX15 orthologs might be of evolutionary relevance. However, during the past 15 years a large number of mammalian genomes have completely been sequenced and thus, it was possible to extract the primary structures of many mammalian ALOX15 orthologs. Based on sequence information of some 60 mammalian ALOX15 orthologs and on functional data of some 30 enzymes the Evolutionary Hypothesis of ALOX15 Specificity (Borngräber et al., 1999; Ivanov et al., 2010) was introduced. This hypothesis suggested that ALOX15 orthologs of mammals ranked in evolution above gibbons express AA 15-lipoxygenating enzymes (Adel et al., 2016a; Kuhn et al., 2018). In contrast, ALOX15 orthologs of mammals ranked below gibbons express AA 12-lipoxygenating enzymes. The ALOX15 of *N. leucogenys* representing gibbons exhibits a pronounced dual reaction specificity since similar amounts of 15- and 12-HpETE were formed (Adel et al., 2016a; Kuhn et al., 2018). In other words, lower mammals including lower primates such as baboons and rhesus monkeys express AA 12-lipoxygenating ALOX15 orthologs (Johannesson et al., 2010; Adel et al., 2016b) but highly developed primates including extinct and extant human subspecies (Sigal et al., 1990; Johannesson et al., 2010; Vogel et al., 2010; Chaitidis et al., 2013; Adel et al., 2015) express AA 15-lipoxygenating enzymes. There are, however, exceptions from this rule. For instance, rabbits (Bryant et al., 1982) and kangaroo rats (Kozlov et al., 2019), which are ranked in evolution below gibbons, express AA 15-lipoxygenating ALOX15 orthologs.

When we started the work described in this paper, the Triad Concept and the Evolutionary Hypothesis were based on sequence data of some 60 stochastically selected mammalian ALOX15 orthologs and about half of them were functionally characterized. Unfortunately, these enzymes have not been purposefully selected because of the evolutionary position of the corresponding animals but rather by chance or by non-evolutionary criteria. Thus, the Evolutionary Hypothesis and the Triad Concept were based on a limited number of coincidental experimental data. To put the two hypotheses on a much broader and more reliable experimental basis, we first extracted the ALOX15 cDNA sequences of 152 mammals representing all major subclades of *Prototheria*, *Metatheria* and *Eutheria* and concluded their reaction specificities employing the Triad Concept as predictive tool (Borngräber et al., 1999; Vogel et al., 2010; Ivanov et al., 2015). To proof these predictions and hence to test the predictive value of the Triad Concept, we expressed 44 novel mammalian ALOX15 orthologs (Supplementary Figure S1) as recombinant N-terminal his-tag proteins and quantified their reaction specificity experimentally. Our data indicate that the Triad Concept is applicable to all mammalian ALOX15 orthologs and that the vast majority (>95%) of these enzymes follow the Evolutionary Hypothesis.

## Materials and Methods

### Chemicals

The chemicals used for this study were obtained from the following sources: Arachidonic acid (AA) and authentic HPLC standards of HETE-isomers (15*S*-HETE, 15*S/R*-HETE, 12*S/R*-HETE, 12*S*-HETE, 5*S/R*-HETE, 5*S*-HETE) from Cayman Chem (distributed by Biomol GmbH, Hamburg, Germany); acetic acid from Carl Roth GmbH (Karlsruhe, Germany); sodium borohydride from Life Technologies, Inc (Eggenstein, Germany); isopropyl-β-thiogalactopyranoside (IPTG) from Carl Roth GmbH (Karlsruhe, Germany); restriction enzymes from ThermoFisher (Schwerte, Germany); the *E. coli* strain Rosetta2 DE3 pLysS from Novagen (Merck-Millipore, Darmstadt, Germany); HEK293 cells from the German Collection of Microorganisms and Cell Culture GmbH (DSMZ, Braunschweig, Germany). The Bac-to-Bac^∗^ baculovirus expression system was purchased from Invitrogen Life Technologies (ThermoFisher, Schwerte, Germany). Oligonucleotide synthesis was performed at BioTez Berlin Buch GmbH (Berlin, Germany). Nucleic acid sequencing was carried out at Eurofins MWG Operon (Ebersberg, Germany). HPLC grade methanol, acetonitrile, n-hexane, 2-propanol and water were from Fisher Scientific (New Hampshire, United States).

### Database Searches

The amino acid sequences of different mammalian ALOX15 orthologs were extracted from the NCBI database (https://www.ncbi.nlmn.nih.gov). For those mammals, for which no complete ALOX15 sequence was present, we employed a different screening strategy. Initially, we downloaded all genomic sequence reads for a particular species from the NCBI assembly database (https://www.ncbi.nlm.nih.gov/assembly/). Next, we assembled these reads to a searchable genomic database using the makeblastdb method of the BLAST 2.10.0 + standalone program. All obtained individual genomic databases showed a >30 genome coverage. These individual genomic databases were finally screened for ALOX15-like sequences using the human ALOX15 gene as probe. Positive hits were then analyzed with the DNASTAR program (DNAS INC., Madison, United States), and the complete ALOX15 amino acid sequence for each species was extracted. To make the ALOX15 sequences available for the scientific community the identified ALOX15 sequences were deposited in the GenBank Third Party Annotation database (https://www.ncbi.nlm.nih.gov/bioproject/) and can be retrieved using the accession number provided in Supplementary Table S1.

### Cloning of Mammalian ALOX15 Orthologs

To test the functionality of selected ALOX15 orthologs, we first extracted the cDNAs from the genomic sequence. A *Sal*I restriction site was introduced immediately upstream of the start codon and the starting ATG encoding for Met was kept unaltered. A *Hind*III recognition sequence was designed immediately downstream the stop codon and internal *Hind*III and *Sal*I sites were eliminated by silent nucleotide exchanges. The cDNA sequences were optimized for bacterial expression and the constructs were chemically synthesized (Biocat GmbH, Heidelberg, Germany). For prokaryotic expression, the construct was excised from the synthesizing vector (pUC57) and cloned into the expression plasmid pET28b (Novagen/Merck, Darmstadt, Germany). Recombinant expression plasmids were tested for the ALOX15 inserts by *Sal*I + *Hind*III digestion and the final expression constructs were sequenced (Eurofins Genomics Germany GmbH, Ebersberg, Germany).

### Bacterial Expression of ALOX15 Orthologs

Bacterial expression of recombinant ALOX15 orthologs were performed as described in (Aldrovandi et al., 2018) for the ALOX15 isoform of *P. aeruginosa*. In brief, competent *E. coli* cells (strain Rosetta two DE3 pLysS) were transformed with 50–100 ng of the recombinant expression plasmid and the cells were grown overnight on kanamycin/chloramphenicol containing agar plates. An isolated bacterial clone was picked and two 1 ml bacterial liquid cultures (LB medium with 50 μg/ml kanamycin and 35 μg/ml chloramphenicol) were grown for 6–8 h at 37°C under agitation at 180 rpm. An appropriate volume of the pre-cultures was then added to 50 ml of sterile culture medium (ENPRESSO B kit, Enpresso GmbH, Berlin, Germany) in ultra-yield culture flasks (Thomson Instrument Company, Oceanside, United States) containing kanamycin (50 μg/ml) and chloramphenicol (35 μg/ml) as antibiotics to achieve an OD_600_ of 0.1–0.15. The cells were grown overnight at 30°C and 250 rpm. After the OD_600_ had reached values above 5, expression of the recombinant proteins was induced by the addition of 1 mM (final concentration) IPTG and booster tablets as well as reagent A according to the instructions of the vendor (ENPRESSO B kit). Then the cultures were maintained at 22°C for 18 h at 230–250 rpm agitation. Bacteria were harvested, the resulting pellet was reconstituted in a total volume of 5 ml PBS and bacteria were lyzed by sonication (digital sonifier, W‐250D Microtip Model 102, 50% maximal sonication amplitude; Branson Ultraschall, Fürth, Germany). Cell debris was spun down (15 min, 15,000 × g, 4°C) and the lysate supernatants were employed as enzyme source.

### Eukaryotic Expression of ALOX15 Orthologs in HEK293 Cells

ALOX15 orthologs, for which only low expression levels were reached in *E. coli*, were expressed in HEK293 cells. For this purpose, the coding sequence including the starting methionine and the his-tag epitope of the prokaryotic expression plasmid pET28b (+) was excised employing *XbaI* and *HindIII* restriction endonucleases. After linearization of the eukaryotic expression vector pcDNA 3.1 (-) (Thermo Fisher Scientific, Schwerte, Germany) with the same enzymes the 2 kbp restriction fragment was ligated into the linearized vector using the Rapid DNA Ligation Kit (Thermo Fisher Scientific). The recombinant plasmid was amplified in *E. coli XL-1 Blue* competent cells (Thermo Fisher Scientific, Schwerte, Germany). HEK239 cells were seeded at a density of about 4×10^5^ cells per 2 ml in DMEM medium (4.5 g/L glucose, l-glutamine, 1 mM sodium pyruvate, 3.7 g/L NaHCO3 containing 10% fetal calf serum) into each well of a 6-well plate (Sarstedt, Nümbrecht, Germany). Cells were allowed to attach to the plastic dishes for 24 h prior to transfection. Cells were transfected using the TransIT-LT1 transfection kit (Mirrus bio, Madison, United States). For this purpose, 2 μg of plasmid DNA were mixed with 6 μL TransIT-LT1 in 194 μL OptiMEM (Life Technologies, Inc., Eggenstein, Germany) and transfection complexes were allowed to form according to the manufacturer’s protocol. Finally, the transfection mixture was added to each well and the cells were incubated with the transfection mixture at 37°C and 5% CO_2_. After 48 h incubation, the cells were washed with PBS, spun down (1,000×*g* for 5 min) and the cell pellet was reconstituted in 0.5 ml of PBS. Cells were disrupted by sonication using an UP50H tip sonifier (Hielscher Ultrasound Technology, Teltow, Germany). Cell debris was spun down (15,000×*g*, 20 min, 4°C) and the lysis supernatant was used for activity assays, protein quantification, SDS-PAGE and Western blot analyses.

### Expression of ALOX15 Orthologs in Sf9 Insect Cells

ALOX15 orthologs, which could neither be effectively expressed in *E. coli* nor in HEK293 cells were expressed as N-terminal recombinant hexa-his-tag fusion proteins in Sf9 insect cells. For this purpose, the ALOX15 coding region was excised from the recombinant bacterial expression plasmids and ligated into the pFastBac HT vector. The bacmids and the recombinant baculoviruses were generated according to the manufacturer’s instructions (Bac-to-Bac^∗^ Baculovirus Expression System, Invitrogen Life Technologies/ThermoFisher, Schwerte, Germany). Protein expression was initiated in *Sf9* cells (ATCC^∗^ CRL-1711) cultures using the Insect XPRESS Medium (Biozym Scientific GmbH, Hessisch Oldendorf, Germany) supplemented with 4 mM glutamine and 0.5% FCS. The Cells were infected with a DOI of one and subsequently incubated at 27°C and 120–130 rpm on an agitation platform. After 72 h of incubation (30% dead cells), the cells were harvested by centrifugation, lysed by sonication and the lysate supernatant were used as enzyme source.

### SDS-PAGE and Western Blot

To test ALOX15 expression aliquots (2–20 µL) of the lysis supernatants were analyzed by SDS-PAGE on a 7.5% polyacrylamide gel. Separated proteins were transferred to a 0.45 µm nitrocellulose membrane (Thermo Scientific GmbH, Schwerte, Germany) by a wet blotting method (ProSieve Ex Western Blot Transfer Buffer 10x, Biozym Scientific GmbH, Hessisch-Oldendorf, Germany). The membranes were blocked with blocking solution (10x BlueBlock PF for Blotting, SERVA Electrophoresis GmbH, Heidelberg, Germany), washed three times with PBS/0.3% TWEEN 20 and were finally incubated with an anti-His-HRP antibody (Miltenyi Biotec GmbH, Bergisch Gladbach, Germany) for 1–2 h at room temperature. After several steps of washing, the membrane was stained using the SERVALight Polaris CL HRP WB Substrate Kit (SERVA Electrophoresis GmbH, Heidelberg, Germany) for 5 min at room temperature. Chemiluminescence was quantified using the FUJIFILM Luminescent Image Analyzer LAS-1000plus (Fujifilm Europe GmbH, Düsseldorf, Germany).

### Site Directed Mutagenesis

Site directed mutagenesis was carried out using the PfuUltra II Hotstart PCR Master Mix kit (Agilent Technologies Germany GmbH & Co. KG, Waldbronn, Germany). For this purpose, 10–50 ng plasmid-DNA were incubated with the specific primer pair (1 µL of 5 µM solution each) and 12.5 µL Pfu UltraI II Hot Start PCR Master Mix in a total volume of 25 µL adjusted with sterile water. The PCR protocol was as follows: 95°C for 1 min initial denaturation, cycle: 30 s at 95°C (denaturation phase), then 60 s at 55°C (annealing phase) followed by the synthesis phase (10 min at 68°C). This cycle was repeated 18 times. Subsequently the parent DNA was digested with 1 µL DpnI (Thermo Scientific, Schwerte, Germany) for 30 min and the digestion was concluded by incubating the samples at 80°C for 10min 8 µL of the PCR sample were used for transformation of competent *E. coli* XL-1 Blue cells (Agilent Technologies Inc., Santa Clara, United States). After incubation for 30 min on ice, the cells were heat shocked for 45 s at 42°C, kept on ice for 2 min and then 400 µL SOC Medium was added. After 1 h incubation at 37°C, the cells were plated on an LB-agar plate supplemented with 50 μg/ml kanamycin (for pET28b) or 100 μg/ml ampicillin (for pcDNA 3.1 or pFastBac HT) and incubated overnight at 37°C. Four isolated growing clones were selected for liquid culture in 2 ml LB-Medium and plasmid DNA was prepared using the NucleoSpin Plasmid kit (Macherey & Nagel, Düren, Germany). One of these clones was selected for sequencing (Eurofins Genomics Germany GmbH, Ebersberg, Germany).

### Activity Assays

To assay the catalytic activity of the recombinant enzymes, variable amounts of the enzyme preparations (bacterial lysate supernatants, HEK293 lysate supernatants, Sf9 insect cell supernatants) were added to 0.5 ml of PBS containing the different substrates (AA, EPA) at a final concentration of 100 µM. After 5 min of incubation, the hydroperoxy compounds formed were reduced to the corresponding alcohols (1 mg of solid sodium borohydride), the samples were acidified (35 µL of acetic acid), proteins were precipitated (addition of 0.5 ml of ice-cold acetonitrile) and precipitated proteins were removed by centrifugation. Aliquots of the protein-free supernatants (50–300 µL) were injected to RP-HPC analyses. A Shimadzu instrument equipped with a Hewlett Packard diode array detector 1040 A was used and metabolites were separated on a Nucleodur C18 Gravity column (Macherey-Nagel, Düren, Germany; 250 × 4 mm, 5 μm particle size) coupled with a guard column (8 × 4 mm, 5 μm particle size). A solvent system consisting of acetonitrile:water:acetic acid (70:30:0.1, by vol) was employed at a flow rate of 1 ml/min and analytes were eluted isocratically at 25°C. For quantification the chromatographic scale was calibrated injecting known amounts of 15-HETE (six-point calibration curve).

### Statistics

Statistic evaluation of the activity data and quantification of the patterns of AA oxygenation products was carried out with the two-sided Student’s t-test using the Microsoft Excel software package (Excel 2016). Numeric *p*-values <0.05 were considered statistically significant.

## Results

### ALOX15 Genes are Absent in the Genomes of Extant *Prototheria*

**FIGURE 1:**
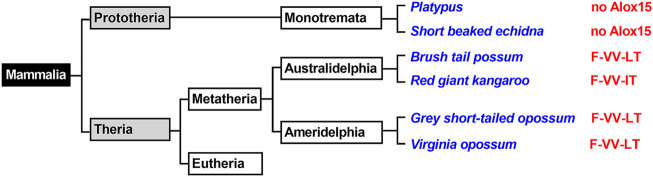
Simplified classification of mammals and presence of ALOX15 orthologs in *Metatheria*. Mammals can be classified in *Prototheria* (ancient mammals) and *Theria,* which may further be subclassified into *Metatheria* and *Eutheria*. *Monotremata* represent extant *Prototheria* and the genomes of platypus and of the short beaked echidna have completely been sequenced. No *ALOX15* genes have been detected in these genomes. *Metatheria* can be subclassified into *Ameridelphia* and *Australidelphia* and we selected the ALOX15 orthologs of two *Ameridelphia* (grey short-tailed opossum, Virginia opossum) and two *Australidelphia* (common brush-tail possum, red giant kangaroo) for functional characterization. Subclassification of *Eutheria* is given in Figures 3–6. The amino acids occupying the triad positions (amino acids aligning with Phe353-Ile418 + Met419-Ile593 + Thr594 of human ALOX15 in dual amino acid sequence alignments), are given by the red letters. The one letter code for amino acids was employed.

Prototheria, Metatheria and Eutheria refer to the three clades of mammals (Figure 1), for which we have currently extant representatives (Burgin et al., 2018)**.** Each of these three phyla may be classified as crown-assemblage that involves extant and extinct species (Budd and Jensen, 2000). Prototheria are the most ancient mammals and *Monotremata* (platypus and four species of echidna) constitute the only extant representatives. Today, all of these mammals are indigenous to Australia and/or New Guinea but there is evidence that they once also occurred in South America (Rauhut et al., 2002)**.** Monotremes lay eggs rather than bearing live young but like all mammals, female monotremes nurse their progeny with milk they produce. Although the genomes of representative monotremes have been sequenced at high quality, the presence or absence of enzymes involved in AA metabolism have not been explored. To fill this gap we screened the genomic databases of platypus and of the short-beaked echidna for ALOX15 genes but could not find corresponding sequences (Figure 1). On the other hand, we found functional genes encoding for all other human ALOX-paralogs and this data is summarized in Table 1. All of these genes carry full-length open reading frames and thus, they are likely to encode for functional ALOX isoforms.

**TABLE 1:** Functional ALOX genes detected in the genomes of *Prototheria*.

Human ALOX isoform	Degree of Amino Acid Identity (%)	
	*Tachyglossus aculeatus* (short baked echidna)	*Ornithorhynchus anatinus* (platypus)
ALOX15	not present	not present
ALOX15B	64.2	59.9; 64.2^a^
ALOX12	65.5	66.61
ALOX12B	78.3	78.14
ALOX5	87.1	87.39
ALOXE3	73.6	74.37

The genomes of the two *Prototheria* were analyzed as described in the Material and Methods section and screened for ALOX-isoforms using the genes of the corresponding human orthologs as probes. The degrees of amino acid conservation of the prototherian ALOX isoforms with the human orthologs are given. Interestingly, no orthologs for the mouse Aloxe12 were detected in these species.

a Two highly similar ALOX15B genes were detected in this species.

### ALOX15 Orthologs are Expressed in *Metatheria*

**FIGURE 2:**
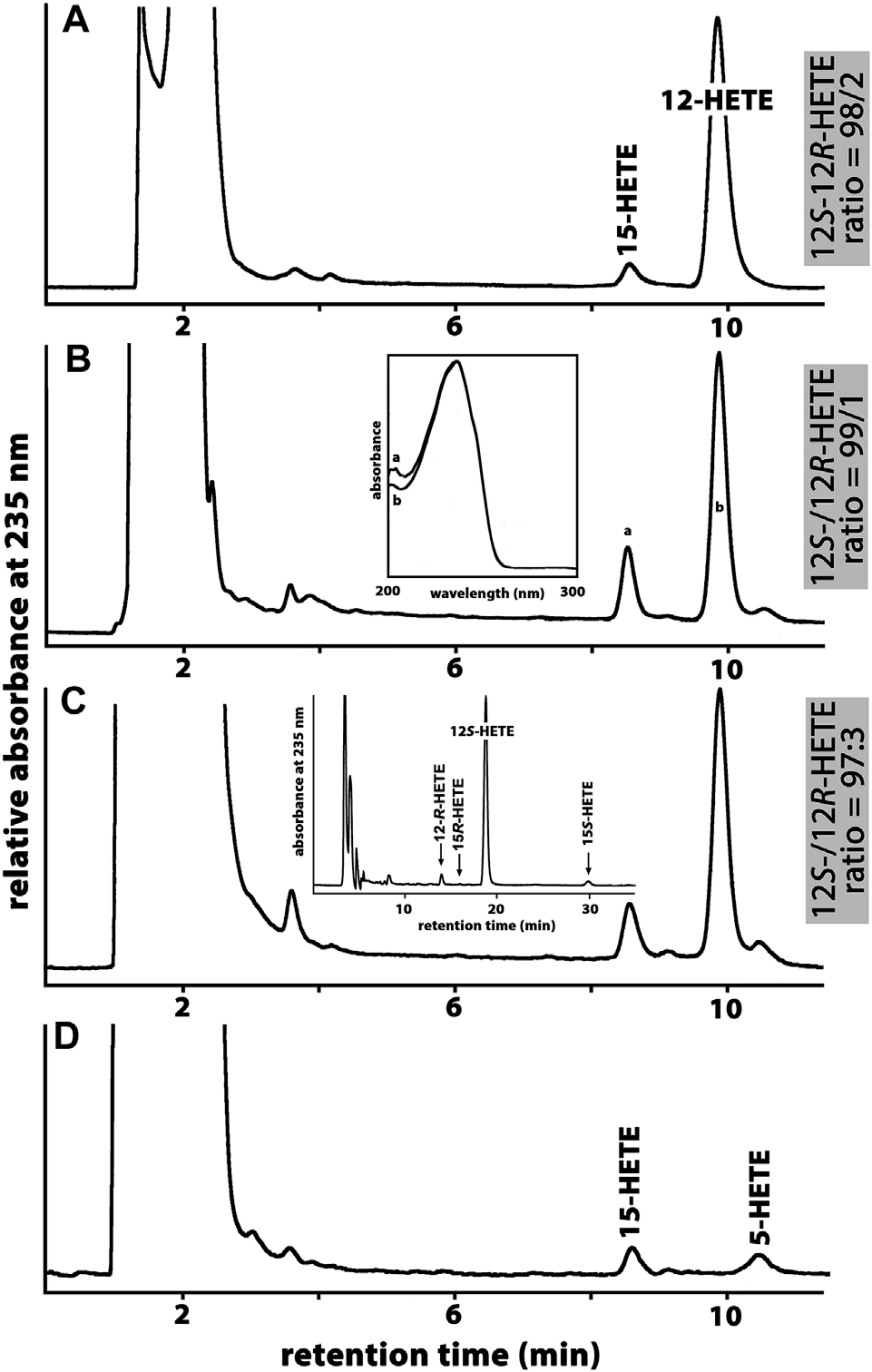
Functional characterization of novel metatherian ALOX15 orthologs. The nucleotide sequences of the cDNAs of metatherian ALOX15 orthologs were extracted from the genomic databases and the corresponding proteins were expressed as recombinant N-terminal hexa-his-tag fusion proteins. Cellular lysate supernatants were used as enzyme source. **(A)** RP-HPLC analysis of the AA oxygenation products formed by the ALOX15 ortholog of the common brushtail possum. **(B)** RP-HPLC analysis of the AA oxygenation products formed by the ALOX15 ortholog of the red giant kangaroo. Inset: UV-spectra of the conjugated dienes taken during the chromatographic run at the time points indicated by the small letters. **(C)** RP-HPLC analysis of the AA oxygenation products formed by the ALOX15 ortholog of the Virginia opossum. Inset: NP/CP-HPLC analysis indicating the enantiomer ratio of the major oxygenation product (12*S*-/12*R*-HETE). **(D)** RP-HPLC analysis of the AA oxygenation products of the no-enzyme control (no lysate supernatant).

All mammals that are more closely related to marsupials than to placentals have been classified as *Metatheria*. This clade involves extant American (*Ameridelphia*) and Australian (*Australidelphia*) marsupials but also a number of extinct non-marsupial relatives (Nilsson et al., 2010)*.* For this study*, we* screened the public genomic databases (https://www.ncbi.nlmn.nih.gov) and reconstructed based on multiple genomic sequence reads the ALOX15 cDNAs of eight different *Metatheria* representing both, *Australidelphia* and *Ameridelphia* (Supplementary Table S1). Although one sequence was incomplete (koala), analysis of the triad determinants indicated the presence of a bulky Phe(F) at the Borngraber-1 position [Phe(F)353)] and a Val(V)+Val(V)/Leu(L) motif at the Sloane positions [Val(V)418 + Val(V) (Leu(L))419]. Based on the Triad Concept these sequence data suggested AA 12-lipoxygenating ALOX15 orthologs for all metatherian ALOX15 orthologs. To test this prediction, we selected the ALOX15 orthologs of two *Ameridelphia* [grey short-tailed opossum (*Monodelphis domestica*), Virginia opossum (*Didelphis virginiana*)] and two *Australidelphia* [common brush-tail possum (*Trichosurus vulpecula*), red giant kangaroo (*Macropus rufus*)] for functional characterization. For the ALOX15 ortholog of the grey short-tailed opossum, AA 12S-lipoxygenation has previously been reported (Kozlov et al., 2019) and we confirmed this result in the present study (data not shown). When we incubated the recombinant ALOX15 orthologs of the common brushtail possum (Figure 2A), the red giant kangaroo (Figure 2B) and the Virginia opossum (Figure 2C) with AA we observed dominant formation of conjugated dienes (UV-spectra of these compounds are given in the inset to Figure 2B) co-migrating in RP-HPLC with an authentic standard of 12*S*-HETE. 15*S*-HETE was formed in smaller quantities but we also observed trace amounts of 5-HETE. Since similar amounts of 5-HETE were found in the no-enzyme control incubations, it must be concluded that 5-HETE was not formed by the recombinant enzyme. In addition, to the 5-HETE we observed much smaller amounts of 12- and 15-HETE in the no-enzyme control incubations. For these controls we employed bacterial lysate supernatants prepared from *E. coli* cells that were transformed with the non-recombinant (empty) expression plasmid (Figure 2D).

Since under our analytical conditions 12- and 8-HETE were not reliably separated and since the chirality of the products could not be deduced from the RP-HPLC chromatograms, we prepared the major conjugated dienes (12- and 15-HETE) by RP-HPLC and further analyzed these products by combined normal phase/chiral phase HPLC (NP/CP-HPLC). In this chromatographic system, 12-HETE and 8-HETE were separated and in addition, the 12-HETE, 8-HETE and 15-HETE enantiomers were well resolved (inset to Figure 2C). Detailed quantification of the product patterns of the metatherian ALOX15 orthologs is given in Table 2 and these data indicated that 12*S*-HETE was the dominant AA oxygenation product formed by all metatherian ALOX15 orthologs. It should be stressed at this point that a single representative of metatheria ALOX15 (grey short-tailed opossum AA) has been characterized before (Kozlov et al., 2019) but here we expressed and functionally characterized three additional metatherian ALOX15 orthologs and seven different triad mutants of these enzymes.

**TABLE 2:** Reaction specificity of recombinant *metatherian* and *eutherian* ALOX15 orthologs expressed in this study.

Clade	Suborder		Species	15-HETE	12-HETE	S/R-Ratio	No.
*Metatheria*	*Australidelphia*		Brushtail possum	14.6 ± 0.4	**85.4 ± 0.4**	97/3	1
			Red giant kangaroo	2.0 ± 1.2	**98.0 ± 1.2**	98/2	2
	*Ameridelphia*		Gray short-tailed opossum	10.0 ± 0.5	**90.0 ± 0.5**	100/0	3
			Virginia opossum	2.7 ± 1.4	**97.3 ± 1.4**	87/13	4
*Eutheria*	*Laurasiatheria*		Chinese pangolin	6.5 ± 0.4	**93.5 ± 0.4**	98/2	5
			Polar bear	7.5 ± 0.0	**92.5 ± 0.0**	96/4	6
			Giant panda	4.4 ± 5.1	**95.6 ± 5.1**	97/3	7
			Dingo	0.2 ± 0.2	**99.8 ± 0.2**	100/0	8
			Meerkat	0.3 ± 0.2	**99.7 ± 0.2**	99/1	9
			Asian palm civet	0.0 ± 0.0	**100.0 ± 0.0**	97/3	10
			Fossa	0.0 ± 0.0	**100.0 ± 0.0**	99/1	11
			Spotted hyena	0.0 ± 0.0	**100.0 ± 0.0**	98/2	12
			Bison	6.1 ± 0.5	**93.9 ± 0.5**	99/1	13
			Red deer	0.8 ± 0.0	**99.2 ± 0.0**	98/2	14
			Bactrian camel	0.0 ± 0.0	**100.0 ± 0.0**	99/1	15
			Chinese river dolphin	8.0 ± 1.4	**92.0 ± 1.4**	92/8	16
			Humpback whale	0.4 ± 0.4	**99.6 ± 0.4**	99/1	17
			White rhinoceros	4.8 ± 0.2	**95.2 ± 0.2**	98/2	18
			Common shrew	0.0 ± 0.0	**100.0 ± 0.0**	99/1	19
			Stare nosed mole	5.1 ± 0.5	**94.9 ± 0.5**	100/0	20
			Large flying fox	4.6 ± 0.4	**95.4 ± 0.4**	98/2	21
	*Xenarthra*		Collared anteater	**84.2 ± 0.1**	15.8 ± 0.1	96/4	22
			Giant anteater	**88.8 ± 0.04**	11.2 ± 0.04	98/2	23
			Screaming hairy armadillo	15.5 ± 0.3	**84.5 ± 0.3**	96/4	24
	*Afrotheria*		African elephant	6.3 ± 5.5	**93.7 ± 5.5**	100/0	25
			Rock hyrax	1.4 ± 0.3	**98.6 ± 0.3**	99/1	26
			Cape elephant shrew	0.4 ± 0.5	**99.6 ± 0.5**	100/0	27
	*Euarchontoglires*	*Non-Primates*	Mountain hare	**96.8 ± 0.1**	3.2 ± 0.1	100/0	28
			Squinney	0.5 ± 0.6	**99.5 ± 0.6**	99/1	29
			Meadow jumping mouse	**59.0 ± 1.2**	**41.0 ± 1.2**	97/3	30
			African woodland thicket rat	7.9 ± 0.5	**92.1 ± 0.5**	97/3	31
			Black rat	2.6 ± 0.3	**97.4 ± 0.3**	99/1	32
			Hoary bamboo rat	**93.7 ± 0.2**	6.3 ± 0.2	95/5	33
		*Primates*	Greater galago (bushbaby)	0.0 ± 0.0	**100.0 ± 0.0**	100/0	34
			Coquerel’s sifaka	0.0 ± 0.0	**100.0 ± 0.0**	100/0	35
			Philippine tarsier	3.9 ± 0.1	**96.1 ± 0.1**	100/0	36
			Common marmoset	1.2 ± 1.5	**98.8 ± 1.5**	98/2	37
			Squirrel monkey	0.9 ± 2.1	**99.1 ± 2.1**	98/2	38
			Nancy Ma’s night monkey	8.2 ± 0.2	**91.8 ± 0.2**	100/0	39
			Angolan black/white colobus	2.8 ± 2.6	**97.2 ± 2.6**	100/0	40
			Yellow-cheeked gibbon	**73.2 ± 1.2**	**26.8 ± 1.2**	96/4	41
			Silvery gbbon	**92.2 ± 0.1**	7.8 ± 0.1	98/2	42
			Siamang	**91.7 ± 0.1**	8.3 ± 0.1	98/2	43
			Western hoolock gibbon	**91.5 ± 0.5**	8.5 ± 0.5	97/3	44
			Müller’s Gibbon	**92.6 ± 0.1**	7.4 ± 0.1	100/0	45

The recombinant enzymes, which involve the Triad determinants specified in Supplementary Figure S1, were expressed as N-terminal hexa-his-tag fusion proteins and *in vitro* activity assays were carried out. The relative shares of 12-HETE and 15-HETE were quantified by RP-HPLC and the enantiomer ratio (*S*/*R*-ratio) of the major oxygenation products determined by NP/CP-HPLC is given. Major oxygenation products are given in bold face. ALOX15 orthologs violating the Evolutionary Hypothesis of ALOX15 specificity are labeled by grey background.

### Functional ALOX15 Genes are Widely Distributed in the Genomes of *Eutheria*

**FIGURE 3:**
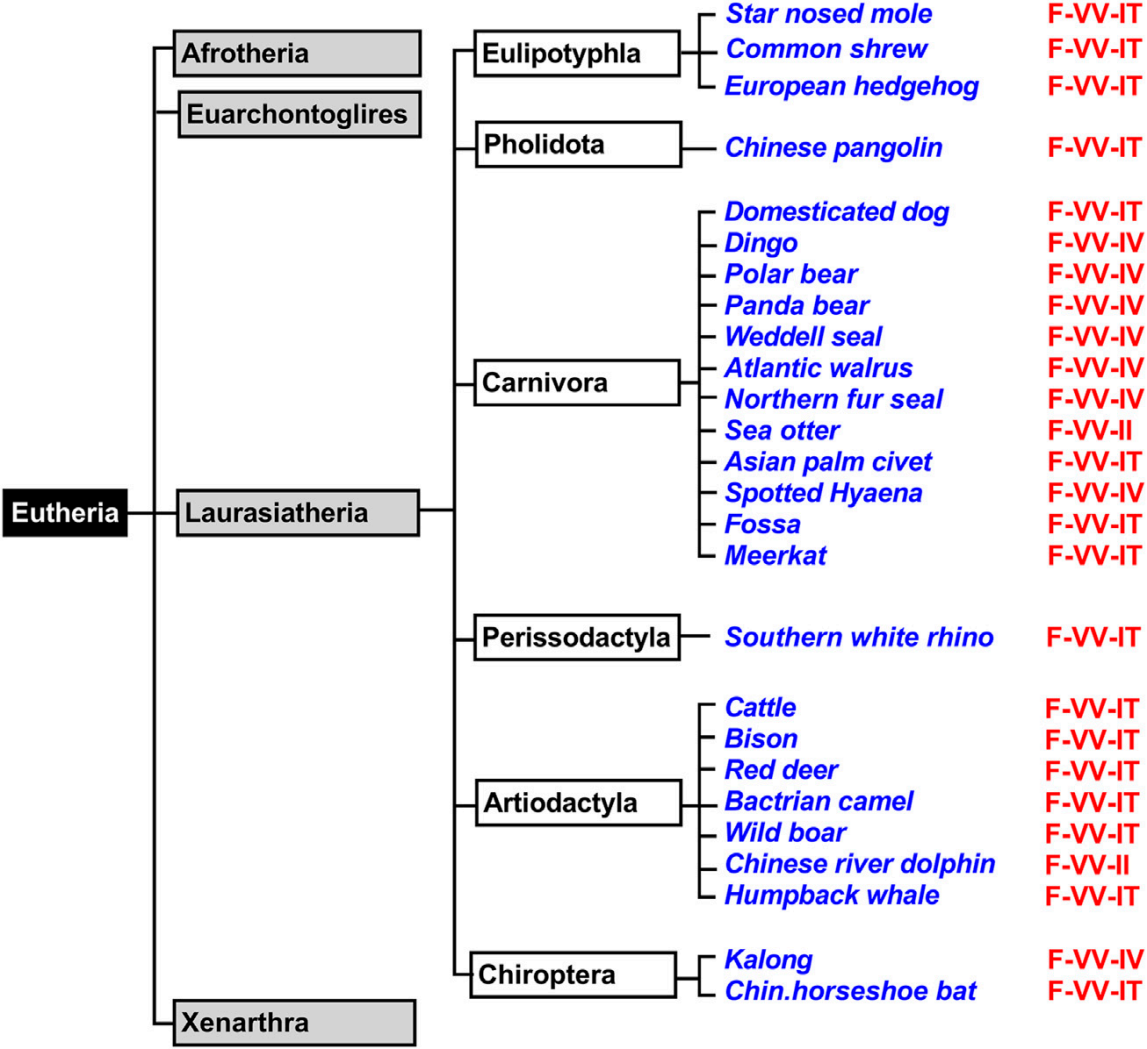
Simplified classification of *eutherian* mammals and subclassification of *Laurasiatheria*. Mammals can be classified into four superorders (*Xenarthra*, *Afrotheria*, *Laurasiatheria*, *Euarchontoglires*) and representative laurasiatherian species, for which complete ALOX15 sequences are available in the databases, have been selected (blue). The triad determinants of the reaction specificity are indicated in red and these data indicate that the Phe353-Val418 + Val419 motif is strongly conserved in all analyzed *Laurasiatheria*.

*Eutheria* form a clade of mammals involving all therian mammals that are more closely related to placentals than to marsupials. All extant *Eutheria* lack epipubic bones, which are present in all living *Prototheria* and *Metatheria* (Reilly and White, 2003). This allows expansion of the abdomen during pregnancy. *Eutheria* can be subclassified in four superorders (*Afrotheria*, *Xenarthra*, *Laurasiatheria*, *Euarchontoglires*) and we first searched the genomic databases of *Laurasiatheria* for ALOX15 orthologs (Figure 3). During this search, we selected 76 complete ALOX15 cDNAs and all of them carried the Phe(F)353, Val(V) (Ala(A))418 + Val(V)419 motif at the Borngraber-1 and the Sloane positions (Supplementary Table S1). Based on the Triad Concept AA 12-lipoxygenation was predicted for the corresponding enzymes.

*Laurasiatheria* can be sub-classified in different taxa and a simplified subclassification scheme of these mammals is given in Figure 3. For functional characterization, we randomly selected laurasiatherian species representing these taxa, expressed their ALOX15 orthologs as recombinant proteins and quantified their reaction specificity experimentally. From Table 2 it can be seen that all randomly selected *Laurasiatheria* (26 different species) express AA 12*S*-lipoxygenating ALOX15 orthologs. It should be stressed at this point that nine out of these 26 different enzymes (Kozlov et al., 2019; Reisch et al., 2021) have been characterized before but 17 of them are novel. Taken together, these data indicate that the Evolutionary Hypothesis is applicable for the ALOX15 orthologs of all *Laurasiatheria*.

**FIGURE 4:**
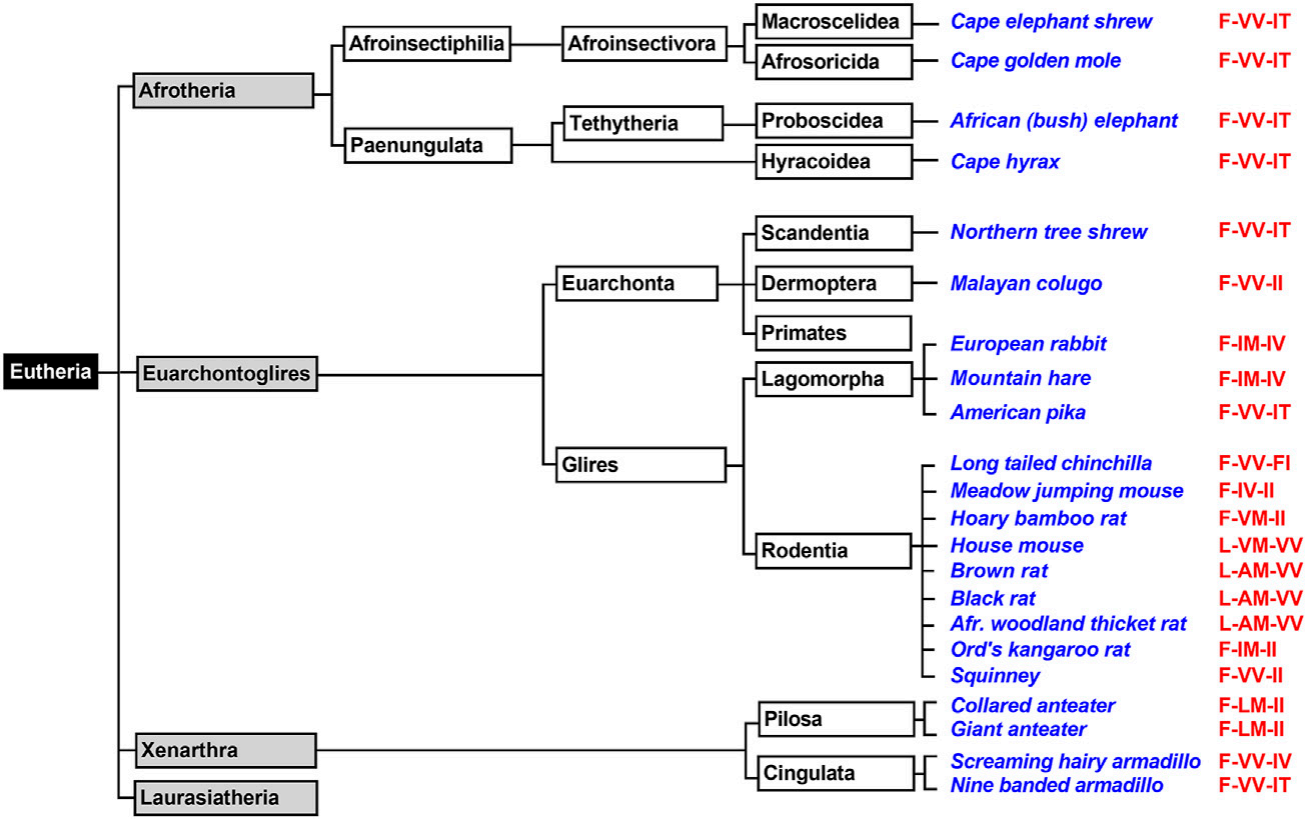
Subclassification of *Xenarthra*, *Afrotheria*, *and Euarchontoglires.* The mammalian superorders *Xenarthra*, *Afrotheria*, *and Euarchontoglires* can be subclassified and representative example species, for which complete ALOX15 sequences are available in the databases, are given in blue. The triad determinants of the reaction specificity are indicated in red. For those species carrying a Leu353-Val(Ala)418 + Met419 motif (house mouse, brown rat) AA 12-lipoxygenating ALOX15 orthologs can be predicted. For those species with a Phe353-Val418 + Val419 (African elephant, cape hyrax) AA 12-lipoxygenation is probable. Phe353-Ile418 + Met419 carrying ALOX15 orthologs (european rabbit, mountain hare, Ord’s kangaroo rat) should express AA 15-lipoxygenating enzymes. For the less frequently occurring triad motifs such as Phe353-Val418 + Met419 (bamboo rat), Phe353-Ile418 + Val419 (meadow jumping mouse) and Phe353-Leu418 + Met419 (collared anteater) reliable predictions of their reaction specificities cannot be made. For such enzymes, experimental proof is required. Subclassification of *Laurasiatheria* is given in Figure 3 and primates are further subclassified in Figure 5.

*Afrotheria* form another mammalian superorder (Figure 4) and this taxon involves species that are either currently living in Africa or are of African origin (Tabuce et al., 2007; Seiffert, 2007). Most groups of afrotherian mammals share little superficial resemblance and their similarities have only become known in recent years because of comparative molecular genetics. Since Africa was an island continent for about 40 million years, laurasian mammals could not migrate into Africa. As consequence, in the frame of convergent evolution, the evolutionary niches occupied by insectivores on the northern continents were filled by insectivorous afrotheres such as elephant shrews, golden moles, and tenrecs. Hyraxes filled the roles of rodents and lagomorphs and elephants took the roles of hippos, camels and rhinos (Tabuce et al., 2007; Stanhope et al., 1998). When we searched the genomes of *Afrotheria,* we found complete ALOX15 sequences for the following species: African elephant, rock hyrax, cape golden mole, lesser hedgehog tenrec and cape elephant shrew (Supplementary Table S1). The corresponding enzymes carry the Phe(F)353, Val(V)418 + Val(V)419 motif at the major triad positions (Supplementary Table S1) and thus, AA 12-lipoxygenating ALOX15 orthologs could be predicted. Unfortunately, except for the cape golden mole (Kozlov et al., 2019), neither of these enzymes has been characterized before. To fill this gap, we selected the enzymes of the African elephant, the rock hyrax and the cape elephant shrew, which represent different afrotherian subclades (Figure 4) and observed dominant AA 12-lipoxygenation for the three enzymes (Table 2). Similar results have previously been reported for the ALOX15 ortholog of the cape golden mole (Kozlov et al., 2019), which represents *Afrosoricida*, a further afrotherian subclade (Figure 4). In conclusion, as *Laurasiatheria* all functionally characterized *Afrotheria* express AA 12-lipoxygenating ALOX15 orthologs and thus, they follow the Evolutionary Hypothesis (Kuhn et al., 2018).

*Xenarthra* form a distinct superorder of placental mammals (Figure 4) and these animals are unique to the Americas. There are more than 30 extant xenarthrian species and the name *Xenarthra* derived from their vertebral joints, which have extra articulations. *Xenarthra* can be subclassified into *Cingulata* and *Pilosa* (Figure 4) and for our studies (Supplementary Table S1), we analyzed the ALOX15 cDNA sequences of two representatives of *Pilosa* [giant anteater (*Myrmecophaga tridactyla*), collared anteater (*Tamandua tetradactyla*)] and two representatives of *Cingulata* [screaming hairy armadillo (*Chaetophractus vellerosus*) and nine banded armadillo (*Dasypus novemcinctus*)]. When we started this project, there was no functional information on ALOX15 orthologs for any xenarthrian mammal. Here we found that the two *Cingulata* species (collared anteater, giant anteater) carry a Phe(F)353-Leu(L)418 + Met(M)419 motif at the major triad positions and thus their ALOX15 orthologs should be AA 15-lipoxygenating enzymes. If this functional conclusion is correct, these species violate the Evolutionary Hypothesis (Kuhn et al., 2018) since these mammals are ranked in evolution below gibbons. In contrast, the two *Pilosa* species (screaming hairy armadillo, nine-banded armadillo) carry the canonical Phe(F)353-Val(V)418 + Val(V)419 motif that was found in all *Afrotheria* and *Laurasiatheria*. Their ALOX15 orthologs are likely to catalyze AA 12-lipoxygenation and thus, they follow the Evolutionary Hypothesis. To test these functional conclusions, we expressed the ALOX15 orthologs of the collared anteater, the giant anteater and the screaming hairy armadillo and characterized their reaction specificity experimentally. From Table 2 it can be seen that as predicted the ALOX15 orthologs of the two anteater species oxygenated AA mainly to 15*S*-HETE. In contrast, 12S-HETE was the major AA oxygenation product of the screaming hairy armadillo ALOX15. Unfortunately, it was not possible to express the ALOX15 ortholog of the nine-banded armadillo since the extracted ALOX15 cDNA was incomplete. Nevertheless, our functional results on the reaction specificity of *Xenarthra* ALOX15 orthologs indicate that the predictions made on the basis of the sequence data were correct.

*Euarchontoglires*, which are also called supraprimates, form a distinct mammalian superorder and this classification is based on retrotransposon markers (Kumar et al., 2013). Unfortunately, no distinctive anatomical features have been recognized that support this classification but there is neither convincing anatomical evidence supporting alternative classification systems. The *Euarchontoglires* taxon split from its *Lausasiatheria* sister taxon about 100 mill years ago during the *Cretaceous* period (O'Leary et al., 2013; Springer et al., 2013; O’Leary et al., 2013). All representatives of these two sister taxons (*Laurasiatheria*, *Euarchontoglires*) originated from a common ancestor and on the basis of the currently available genomic sequences it was suggested that the genome of this ancestor might have consisted of some three billion base pairs (as humans) and that its genomic sequence can be predicted with an accuracy of about 98% (Blanchette et al., 2004)**.** When we searched the genome databases for ALOX15 sequences, we extracted 14 complete sequences of non-primate *Euarchontoglires* ALOX15 orthologs (Figure 4). Half of them have functionally been characterized before (Bryant et al., 1982; Freire-Moar et al., 1995; Kozlov et al., 2019; Watanabe et al., 1993; Schäfer et al., 2020) but the other half is novel. Analyzing these sequences, we found that the majority of the ALOX15 orthologs involved the Phe(F)353-Val(V)418 + Val(V)419 motif at the major triad determinants and thus, AA 12-lipoxygenating ALOX15 orthologs could be predicted (Supplementary Table S1). In addition, there are several species carrying a Leu(L)353-Ala(A) (Val(V))418 + Met(M)419 motif (brown rat, black rat, east European house mouse, Mongolian gerbil) and the Triad Concept also predicts AA 12-lipoxygenation for these enzymes. This is also the case for the AA 12-lipoxygenating ALOX15 ortholog of rabbits (Borngräber et al., 1996; Berger et al., 1998) that carries the Leu(L)353-Ile(I)418 + Met(M)419 motif (Supplementary Table S1). However, among non-primate *Euarchontoglires* we also identified ALOX15 orthologs carrying the Phe(F)353-Ile(I)418 + Met(M)419 (rabbit, mountain hare, Ord’s kangaroo rat) and these data suggest AA 15-lipoxygenating enzymes. Since these species are ranked in evolution below gibbons, they do not follow the Evolutionary Hypothesis. For the hoary bamboo rat (*Rhizomys pruinosus*) and the meadow jumping mouse (*Zapus hudsonius*) we detected unusual sequence motifs [Phe(F)353-Val(V)418 + Met(M)419 and Phe(F)353-Ile(I)418 + Val(V)419, respectively] at the major triad positions and on the basis of these sequence data it was impossible to reliably predict the reaction specificity of the corresponding enzymes (Supplementary Table S1).

For functional characterization, we selected six different *non-primate Euarchontoglires* (Table 2) that have not been characterized before, expressed the ALOX15 orthologs and analysed their reaction specificity of AA oxygenation. The ALOX15 ortholog of the Chinese tree shrew, which represents *Scandentia* (Figure 4), has previously been characterized as AA 12-lipoxygenating enzyme (Schäfer et al., 2020). *Dermoptera* (Figure 4) are represented by the Malayan colugo and its ALOX15 ortholog is also AA 12-lipoxygenating (Kozlov et al., 2019). The European rabbit, the American pika and the mountain hare represent different subfamilies of *Lagomorpha* (Figure 4). Consistent with the Triad Concept the ALOX15 ortholog of the European rabbit (Bryant et al., 1982) is AA 15-lipoxygenating whereas the corresponding enzyme of the American pika is AA 12-lipoxygenating (Kozlov et al., 2019). In this study, we expressed the ALOX15 ortholog of the mountain hare and observed dominant AA 15-lipoxygenation (Table 2). Thus, although rabbits and mountain hares follow the Triad Concept they do not follow the Evolutionary Hypothesis. Finally, we explored the ALOX15 orthologs of several *Rodentia*, which together with *Lagomorpha* form the unranked *Euarchontoglires* subfamily of *Glires* (Figure 4). Long-tailed chinchillas (Kozlov et al., 2019), house mice (Freire-Moar et al., 1995) and brown rats (Watanabe and Haeggstrom, 1993; Pekárová et al., 2015) express AA 12-lipoxygenating ALOX15 orthologs and this data is consistent with both, the Triad Concept and the Evolutionary Hypothesis. Ord’s kangaroo rats express an AA 15-lipoxygenating ALOX15 ortholog (Kozlov et al., 2019). Although the Triad Concept is applicable for this enzyme, it violates the Evolutionary Hypothesis. For this study (Table 2) we functionally characterized the ALOX15 orthologs of six additional rodents (meadow jumping mouse, hoary bamboo rat, squinney, African woodland thicket rat, black rat) and obtained the following results: 1) Squinneys carrying the Phe(F)353-Val(V)418 + Val(V)419 motif, African woodland thicket rats [Leu(L)353-Ala(A)418 + Met(M)419] and black rats [Leu(L)353-Ala(A)418 + Met(M)419] express dominant AA 12-lipoxygenating ALOX15 orthologs (Table 2). These results were predicted by the Triad Concept and the animals follow the Evolutionary Hypothesis of ALOX15 specificity. 2) The ALOX15 ortholog of the hoary bamboo rat, which carries the Phe(F)353-Val(V)418 + Met(M)419 motif (Figure 4, Supplementary Table S1) converted AA predominantly to 15-HETE (Table 2). Although this finding is consistent with the Triad Concept, this animal does not follow the Evolutionary Hypothesis. 3) The ALOX15 of the meadow jumping mouse, which carries the Phe(F)353 + Ile(I)418 + Val(V)419 motif (Figure 4, Supplementary Table S1), converted AA to almost similar amounts of 12- and 15-HETE (Table 2) and thus, exhibits a pronounced dual reaction specificity. This finding is also consistent with the Triad Concept but it partly violates the Evolutionary Hypothesis.

### The Evolutionary Switch From AA 12- to 15-Lipoxygenating ALOX Isoforms Proceeded During Late Primate Evolution

**FIGURE 5:**
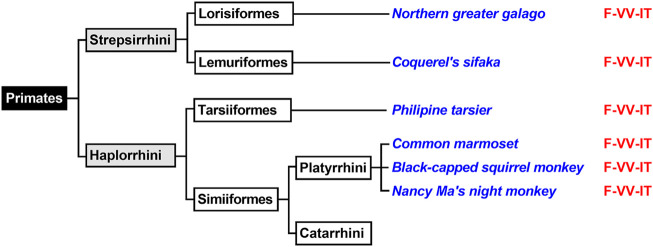
Subclassification of Primates. Primates can be subclassified in two suborders (*Haplorrhini*, *Strepsirrhin*i). Complete ALOX15 cDNA sequences of randomly selected representatives of these two primate suborders (given in blue) were extracted and their triad determinants are given in red. Since all representatives carry the Phe353 + Val418 + Val419 motif, which is canonical for AA 12-lipoxygenating ALOX15 orthologs and thus, AA 12-lipoxygenation was predicted for all these enzymes. Further subclassification of *Catarrhini* is given in Figure 6.

Primates first evolved about 80 million years ago from small terrestrial mammals that lived in the trees of tropical forests. Depending on the classification schemes there are currently 200–400 different extant primate species and novel species are still discovered (Groves, 2001). The mammalian order of primates is subdivided into two suborders (*Strepsirrhini*, *Haplorrhini*) and both suborders can further be subclassified (Figure 5). For this study, we first extracted the ALOX15 cDNA sequences of two randomly selected *Strepsirrhini* representing *Lorisiformes* (northern greater galago) and *Lemuriformis* (coquerel’s sifaka). Then we also extracted the ALOX15 cDNA sequences of selected *Haplorrhini*. *Tarsiiformes* were represented by the Philippine tarsier and *Simiiformes* were represented by three different *Platyrrhini* species (common marmoset, the black-capped squirrel monkey, Nancy Ma’s night monkey). Neither of these ALOX15 orthologs has been characterized before. When we extracted the triad determinants of the ALOX15 orthologs, we found the Phe(F)353 + Val(V)418 + Val(V)419 motif in all cases (Figure 5) and these data predicted AA 12-lipoxygenating enzymes. When we characterized the reaction specificity of the recombinant enzymes experimentally, this prediction was confirmed. The results indicated that the Triad Concept is applicable for these mammalian ALOX15 orthologs and since these species are ranked below gibbons, the enzymes follow the Evolutionary Hypothesis.

**FIGURE 6:**
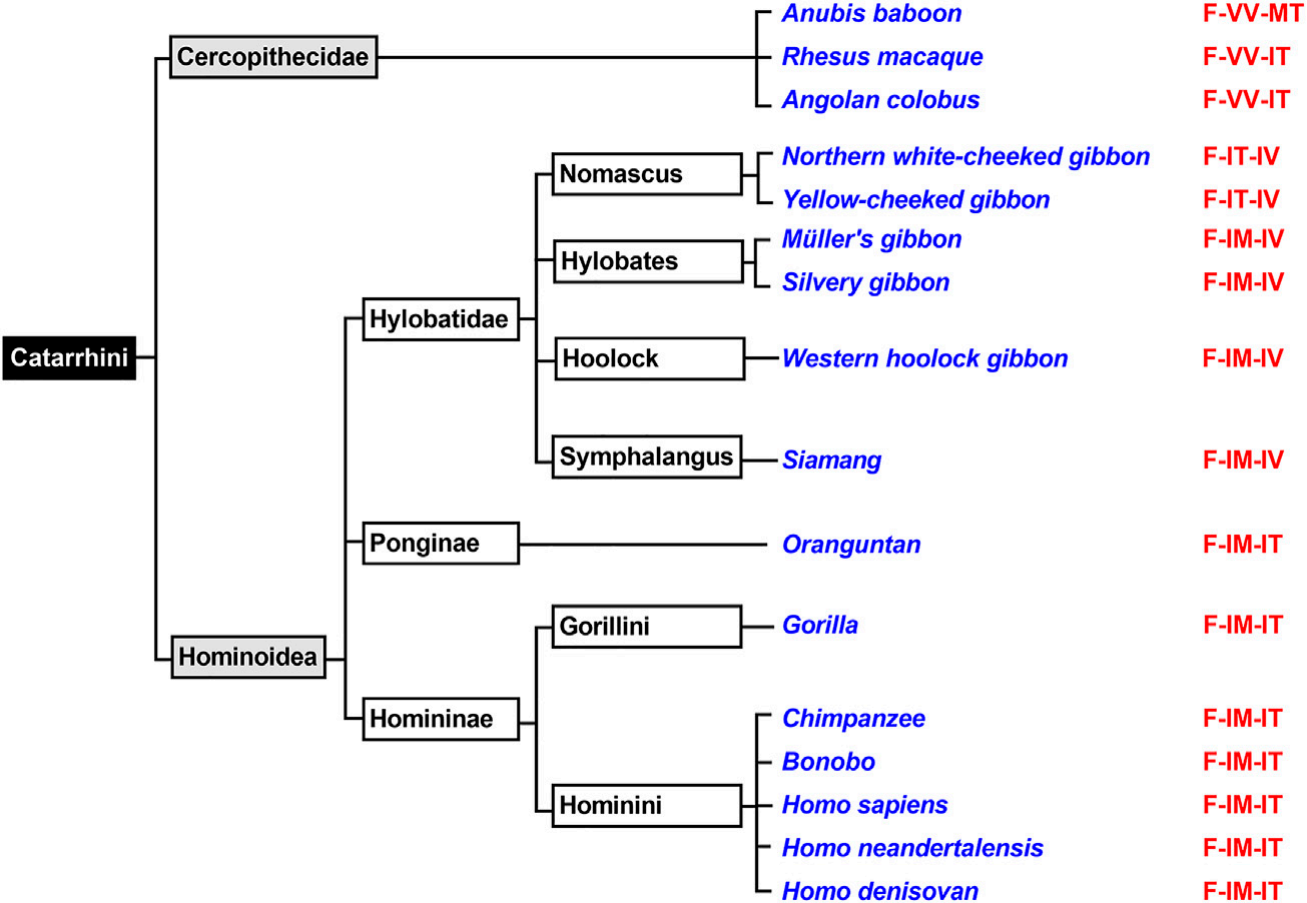
Subclassification of *Catarrhini*. The mammalian parvorder of *Catarrhini* can be subclassified in two families (*Cercopithecidea*, *Hominoidea*). Complete ALOX15 cDNA sequences of randomly selected representatives of these two families (given in blue) were extracted and their triad determinants are given in red. All selected *Cercopithecoidea* carry the Phe353 + Val418 + Val419 motif, which is canonical for AA 12-lipoxygenating ALOX15 orthologs. Thus, AA 12-lipoxygenation was predicted for these enzymes. All selected *Homininae* and *Ponginae* carry the Phe353-Ile418 + Met419 motif and thus, AA 15-lipoxygenation was predicted for the corresponding ALOX orthologs. For Hylobatidae two different triad motifs were extracted. The two *Nomascus* species (Northern white-cheeked gibbon, yellow-cheeked gibbon) carry the Phe353-Ile418 + Thr419 motif and thus are likely to exhibit pronounced dual reaction specificity. In contrast, the two *Hylobates* species (Müller’s gibbon, silvery gibbon) as well as the Western hoolock gibbon and the Siamang carry the Phe353-Ile418 + Met419 motif, which is canonical for AA 15-lipoxygenating ALOX15 orthologs.

The mammalian infraorder of *Simiiformes* (Figure 5) is subdivided into the two parvorders (*Platyrrhini*, *Catarrhini*) and *Catarrhini* can further be classified (Figure 6) into two superfamilies (*Hominoidea*, *Cercopithecidea*). We first analyzed the ALOX15 cDNA sequences of three randomly selected *Cercopithecidea* (anubis baboon, rhesus macaque, angolan colobus) and found the Phe(F)353-Val(V)418 + Val(V)419 motif, which is canonical for AA 12-lipoxygenating ALOX15 orthologs. For the ALOX15 orthologs of anubis baboon (Adel et al., 2016a) and rhesus macaque (Johannesson et al., 2010; Vogel et al., 2010) AA 12-lipoxygenation has previously been shown and we confirmed these results in this study. In addition, we found that the ALOX15 of the Angolan black-and-white colobus does also oxygenate AA predominantly to 12-HETE (Table 2).

The Evolutionary Hypothesis (Kuhn et al., 2018; Adel et al., 2016a) suggested that primates that are ranked above gibbons such as *H. sapiens* (Sigal et al., 1990), extinct human subspecies (Adel et al., 2015), chimpanzee (Adel et al., 2016a), bonobo (Adel et al., 2016a), gorilla (Kozlov et al., 2019) and orangutan (Johannesson et al., 2010; Vogel et al., 2010) carry the Phe(F)353-Ile(I)418 + Met(M)419 motif (Figure 6) and previous functional studies indicated AA 15-lipoxygenating ALOX15 orthologs. In contrast, lower *Catarrhini* such as *Cercopithecidea* (anubis baboon, rhesus macaque) express AA 12-lipoxygenating ALOX15 orthologs (Kuhn et al., 2018; Adel et al., 2016a). *Hylobatidea*, which are ranked in evolution above *Cercopithecidea* but below *Ponginea* (Figure 6), were suggested to function as transition family between AA 12-lipoxygenating and AA 15-lipoxygenating ALOX15 orthologs. This hypothesis was based on the observation that the ALOX15 ortholog of a single *Hylobatidea* species (*Nomascus leucogenys*), which carries the Phe353-Ile418 + Thr419 motif, exhibits a pronounced dual reaction specificity oxygenating AA to similar amounts of 12*S*- and 15*S*-HETE (Adel et al., 2016a). To broaden the experimental basis for the conclusion that *Hylobatidea* represent the transition taxon we first extracted the ALOX15 cDNA sequences of seven additional *Hylobatidea* species (Müller’s gibbon, silvery gibbon, pileated gibbon, lar gibbon, yellow-cheeked gibbon, Western hoolock gibbon, siamang) that have not been characterized before and found that the yellow-cheeked gibbon (*Nomascus gabriellae*) does also carry the Phe(F)353-Ile(I)418 + Thr(T)419 motif. Thus, on the basis of the Triad Concept the two *Nomascus* species (*Nomascus leucogenys, Nomascus gabriellae*) should express ALOX15 orthologs with pronounced dual reaction specificity and our functional data are consistent with this prediction [Table 2, (Adel et al., 2016a)]. On the other hand, the *Hylobates* species (silvery gibbon, pileated gibbon, Lar gibbon, Müller’s gibbon), the western hoolock gibbon and the siamang carry the Phe(F)353-Ile(I)418 + Met(M)419 motif, which is canonical for AA 15-lipoxygenating enzymes. In fact, functional studies on the recombinant enzymes indicated preferential AA 15-lipoxygenation (Table 2). Taken together, these findings indicate that most *Hylobatidea* species express AA 15-lipoxygenating ALOX15 orthologs, as it is the case for the great apes and for humans. However, the *Nomascus* species (*Nomascus leucogenys, Nomascus gabriellae*) exhibit pronounced dual reaction specificities oxygenating AA to similar amounts of 12*S*- and 15*S*-HETE (Supplementary Figure S2). *Ponginea* (orangutans) and *Hominea* (gorillas, chimpanzees, bonobos, humans) carry the Phe(F)353-Ile(I)418 + Met(M)419 motif (Figure 6, Supplementary Table S1), which is canonical for AA 15-lipoxygenating ALOX15 orthologs, and previous functional data are consistent with this prediction (Sigal et al., 1990; Johannesson et al., 2010; Vogel et al., 2010; Adel et al., 2015; Adel et al., 2016a; Kozlov et al., 2019). Taken together the functional data obtained for the nine structurally and functionally characterized *Hylobatidea* species support the previous hypothesis that *Hylobatidea* represent the transition taxon that links AA12-lipoxygenating ALOX15 orthologs (lower evolutionary ranking) with their AA 15-lipoxygenating (higher evolutionary ranking) relatives.

### Site Directed Mutagenesis of the Triad Determinants Alters the Reaction Specificity of all Mammalian ALOX15 Orthologs Independent of Their Evolutionary Ranking

The Triad Concept of ALOX15 specificity suggested that Phe(F)353, Ile(I)418 + Met(M)419 and Ile(I)593 (amino acid numbering for human ALOX15) form the bottom of the substrate binding pocket (Borngräber et al., 1999; Ivanov et al., 2010). If small amino acids are localized at these positions, AA 12-lipoxygenation is possible. In contrast, when bulky residues are located there AA 15-lipoxygenation is preferred. This concept implies that mutagenesis of the less space-filling triad determinants of 12-lipoxygenating ALOX15 orthologs to more bulky residues should alter the reaction specificity favoring AA 15-lipoxygenation. Corresponding experiments have been carried out in the past for some 30 different mammalian ALOX15 orthologs (Sloane et al., 1991; Suzuki et al., 1994; Sloane et al., 1995; Borngräber et al., 1996; Berger et al., 1998; Borngräber et al., 1999; Vogel et al., 2010; Pekárová et al., 2015; Adel et al., 2016a; Kozlov et al., 2019; Schäfer et al., 2020; Reisch et al., 2021) but considering the large number of mammalian species (>6,000) the experimental basis for this hypothesis is still somewhat limited. Moreover, the mammalian ALOX15 orthologs characterized so far do not equally represent the major mammalian subclades. In fact, when we started this project there was hardly any functional information on prototherian and metatherian ALOX15 orthologs. Moreover, most functionally characterized eutherian ALOX15 orthologs originate from *Euarchontoglires*.

When one classifies mammalian ALOX15 orthologs according to the chemistry of their triad determinants three different enzyme subtypes can be distinguished (Supplementary Table S1): 1) ALOX15 orthologs carrying the Phe(F)353-Val(V)418 + Val(V)419 motif at the positions of the major triad determinants. The vast majority of mammalian ALOX15 orthologs belong to this category and these enzymes catalyze AA 12-lipoxygenation (Supplementary Table S1). 2) ALOX15 orthologs carrying the Leu(L) (Ile(I))353-Ile(I) (Ala(A), Gly(G), Val(V))418 + Met(M)419 motif. These enzymes [rabbit (12), house mouse, Gairdner’s shrewmouse, ryukyu mouse, European water vole, African woodland thicket rat, Mongolian gerbil, African grass rat, brown rat, black rat] may also catalyze AA 12-lipoxygenation (Supplementary Table S1). 3) ALOX15 orthologs carrying the Phe353-Ile418 + Met419 motif. This motif is canonical for AA 15-lipoxygenating enzymes and was detected in the ALOX15 orthologs of *H. sapiens*, *H. neandertalensis*, *H. denisovan*, chimpanzee, gorilla, orangutan, rabbit (15), mountain hare, collared anteater, giant anteater (Supplementary Table S1).

**FIGURE 7:**
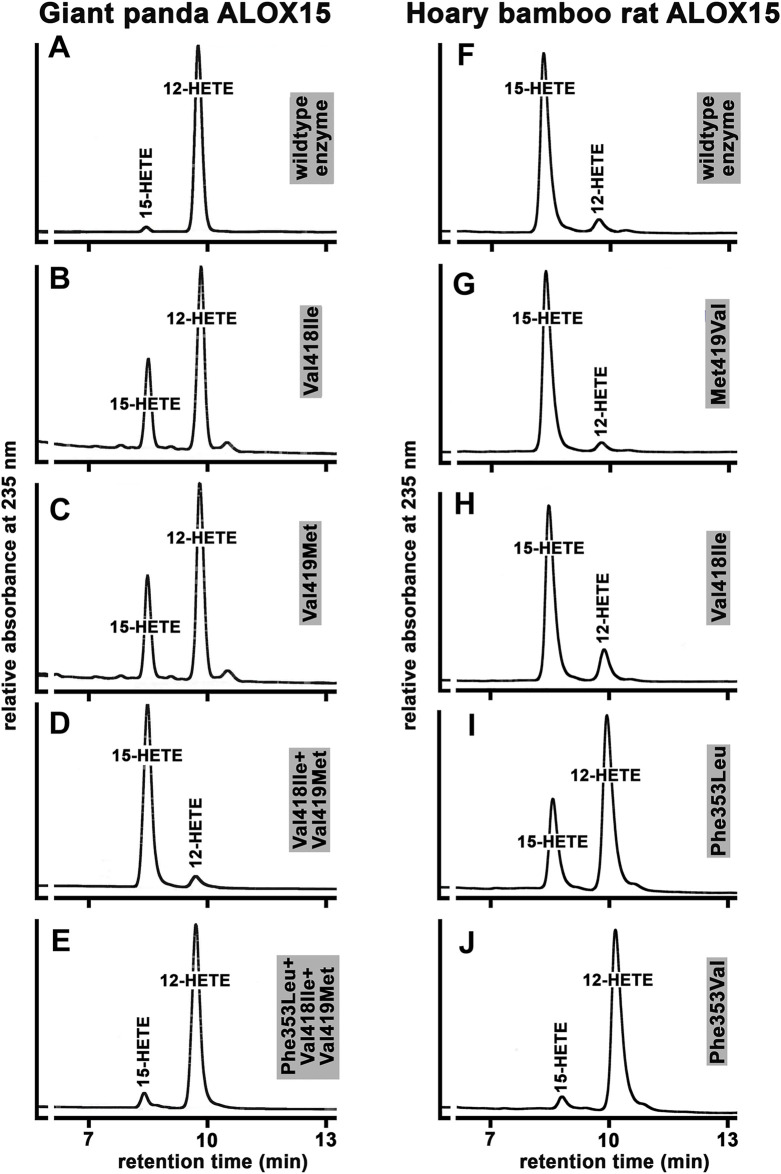
Functional characterization of the wildtype ALOX15 ortholog of the giant panda and the Hoary bamboo rat and of selected mutants of the triad determinants. **(A–E)** RP-HPLC of wild type and mutant Alox15 of the giant panda, **(F–J)** RP-HPLC of wild type and mutant Alox15 of the hoary bamboo rat. The ALOX15 cDNAs of the two mammals were extracted from the genomic databases and the recombinant enzymes were expressed in *E. coli*. Mutagenesis experiments were performed as described in Materials and Methods and the mutant enzyme variants were also expressed. *In vitro* activity assays were carried out using bacterial lysate supernatants and the oxygenation products were analyzed by RP-HPLC. Representative partial chromatograms are shown. Statistic evaluation of the data is given in Supplementary Table S2.

To put the Triad Concept on a broader and more reliable experimental basis we carried out extensive additional mutagenesis studies for a large number of ALOX15 orthologs and the results are summarized in Supplementary Table S2. For better understanding of our mutagenesis strategy, we will explain in detail the experiments carried out with the ALOX15 ortholog of the giant panda and the hoary bamboo rat (Figure 7). The giant panda is classified as *Laurasiatheria* and its ALOX15 ortholog carries the Phe(F)353-Val(V)418 + Val(V)419-Ile(I)593 + Val(V)594 motif (Supplementary Table S1), which is canonical for AA 12-lipoxygenating ALOX15 orthologs. As expected, the wildtype enzyme exhibited a dominant AA 12-lipoxygenating activity (Figure 7A). When we mutated the small Val(V)418 to a more space-filling Ile(I) we found that the mutant enzyme exhibited a dual reaction specificity since 12-HETE and 15-HETE were simultaneously formed at a ratio of about 7:3 (Figure 7B). Then we created the Val(V)419Met(M) mutant and observed a similar product pattern (Figure 7C). Thus, single point mutation of the two Sloane determinants (Val(V)418 + Val(V)419) induced elevated 15-HETE formation although 12-HETE remained the major oxygenation product.

Next, we combined these two mutations [Val(V)418Ile(I)+Val(V)419Met(M), Figure 7D] and observed complete inversion of the reaction specificity. In fact, the Val(V)418Ile + Val(V)419Met(M) double mutant converted AA almost exclusively to 15-HETE and additional NP/CP-HPLC (data not shown) indicated the strong preponderance of the 15S-enantiomer. According to the Triad Concept, this inversion in the reaction specificity should be reversed by additional Phe(F)353Leu(L) exchange. In fact, from Figure 7E it can be seen that the Phe(F)353Leu(L)+Val(V)418Ile(I)+Val(V)419Met(M) triple mutant exhibited a dominant AA 12-lipoxygenating activity. A similar mutagenesis strategy was applied for the AA 12-lipoxygenating ALOX15 orthologs of more than 40 different mammalian species and we always observed a similar trend **(** Supplementary Table S2 **)**. In most cases, Val(V)418Ile(I) exchange was more productive than the Val(V)419Met(M) mutation but for all enzymes tested, the double mutants exhibited the strongest effects (Supplementary Table S2). For all tested ALOX15 orthologs the effect induced by Val(V)418Ile(I) +Val(V)419Met(M) exchange could be at least partially reversed by Phe(F)353Leu(L) (Ala(A)) mutation.

We also performed similar mutagenesis experiments with the ALOX15 ortholog of the hoary bamboo rat. The wildtype enzyme that carries the Phe(F)353-Val(V)418 + Met(M)419 motif exhibits a dominant AA 15-lipoxygenating activity (Figure 7F). When we performed Met(M)419Val(V) exchange to obtain the Phe(F)353-Val(V)418 + Val(V)419 motif, which is canonical for AA12-lipoxygenating ALOX15 orthologs, we surprisingly observed hardly any alteration in the reaction specificity (Figure 7G). Similarly, only minor alterations in the reaction specificity were found for the Val(V)418Ile(I) mutant (Figure 7H), which carries the Phe(F)353-Ile(I)418 + Met(M)419 motif that is canonical for AA 15-lipoxygenating enzymes. These results suggested that this ALOX15 ortholog might violate the Triad Concept since mutagenesis of the Sloane determinants did not induce major specificity alterations. However, when we performed Phe(F)353Leu(L) exchange the reaction specificity was strongly changed in favor of AA 12-lipoxygenation (Figure 7I) and this alteration was even more pronounced for the Phe(F)353Val(V) mutant (Figure 7J). Taken together, these data indicated that mutagenesis of the Sloane determinants did not dramatically alter reaction specificity of this ALOX15 ortholog and that the Borngraber-1 determinant appeared to be more important.

In some AA 12-lipoxygenating ALOX15 orthologs (Supplementary Table S1) a Leu(L) or Ile(I) is located at position 353 [rabbit (12), house mouse, Gairdner’s shrewmouse, Ryukyu mouse, Mongolian gerbil, European water vole, African woodland thicket rat, African grass rat, brown rat, black rat] and the reaction specificity of these ALOX15 orthologs is consistent with the Triad Concept. When we mutated the Leu(L)353 in the mouse, rat and rabbit (12) ALOX15 to a more space filling Phe(F) (Leu(L)353Phe(F)) we observed almost complete conversion of the reaction specificity in favor of AA 15-lipoxygenation (Supplementary Table S2).

Finally, corresponding mutations were carried out for the ALOX15 orthologs carrying the Phe(F)353-Ile(I)418 + Met(M)419 motif [man, chimpanzee, orangutan, rabbit (15), mountain hare, Ord’s kangaroo rat], which is canonical for AA 15-lipoxygenation. When the space-filling residues at Ile(I)418 and Met(M)419 were mutated to less bulky amino acids [Ile(I)418Val(V)/Ala(A)+Met(M)419Val(V)] AA 12-lipoxygenation was favored (Supplementary Table S2). Taken together our mutagenesis studies, which involved expression and functional characterization of 159 mammalian ALOX15 variants that have not been reported before, indicate that all mammalian ALOX15 orthologs tested follow the Triad Concept independent of their evolutionary ranking.

### AA 15-Lipoxygenating ALOX15 Orthologs Exhibit Increased Resolvin E4 Synthase Activities

AA 12- and 15-lipoxygenating ALOX isoforms have been implicated in the biosynthesis of pro-resolving mediators such as lipoxins (Ryan and Godson, 2010) and resolvins (Serhan and Petasis, 2011), which drive inflammatory resolution (Freire and Van Dyke, 2000). In theory, AA 15-lipoxygenating ALOX15 orthologs should exhibit a higher biosynthetic capacity for resolvin E4 than their 12-lipoxygenating counterparts when 5-HEPE is offered as substrate. To test this hypothesis, we quantified the resolvin E4 synthase activity of five randomly selected AA 12- and five AA 15-lipoxygenating ALOX15 orthologs. When normalized to similar AA oxygenase activities we found (Table 3) that AA 15-lipoxygenating ALOX15 orthologs exhibit a 6-fold higher resolvin E4 synthase activity than their AA 12-lipoxygenating counterparts. These data are consistent with the hypothesis that the switch in reaction specificity of ALOX15 orthologs during late primate evolution allows an improved control of the inflammatory reaction, which might confer an evolutionary advantage to primates that are ranked above gibbons. Thus, the evolutionary switch in reaction specificity of ALOX15 orthologs may contribute to optimize the immune response.

**TABLE 3:** Resolvin E4 synthase activity of AA 12- and AA 15-lipoxygenating ALOX15 orthologs.

Species	15-HETE (%)	12-HETE (%)	Relative RvE4 synthase activity (%)^a^
AA 15-lipoxygenating ALOX15 orthologs			
Man	88.0 ± 2.3	12.0 ± 2.3	32.6 ± 5.8
Orangutan	90.5 ± 2.9	9.5 ± 2.9	34.7 ± 3.0
Rabbit	92.3 ± 2.8	7.7 ± 2.8	27.2 ± 8.2
Giant anteater	90.7 ± 5.0	9.3 ± 5.0	20.0 ± 3.8
Bamboo rat	91.4 ± 2.9	8.7 ± 2.9	55.9 ± 15.5
**mean ± SD**	**90.6 ± 3.3**	**9.4 ± 3.3**	**34.2 ± 14.5** ^b^
AA 12-lipoxygenating ALOX15 orthologs			
Rhesus macaque	1.8 ± 1.2	98.2 ± 1.2	4.8 ± 1.9
Polar bear	7.5 ± 2.4	92.5 ± 2.4	5.7 ± 3.0
Kalong	9.6 ± 4.6	90.4 ± 4.6	5.2 ± 1.1
Humpback whale	1.9 ± 1.3	98.2 ± 1.3	6.0 ± 1.9
Star nosed mole	15.9 ± 3.8	84.2 ± 3.8	6.5 ± 0.8
**mean ± SD**	**7.3 ± 6.0**	**92.7 ± 6.0**	**5.6 ± 1.8** ^**#**^

Aliquots of the bacterial lysate supernatant of the different enzyme preparations were incubated for 5 min in PBS with 100 µM arachidonic acid for 5 min or with 20 µM 5S-HEPE for 10 min to quantify the arachidonic acid oxygenase activity and the resolvin E4 (RvE4) synthase activity, respectively. The amounts of 12-HETE+15-HETE (AA oxygenase activity assay) and of 5*S*,15*S*-DiHEPE (resolvin E4 synthase activity assay) formed during the incubation period were quantified by RP-HPLC. The reaction volume was 0.1 ml. The hydroperoxy fatty acids formed were reduced (addition of 1 mg solid sodium borohydride), the samples were acidified, proteins were precipitated with 0.1 ml ice-cold acetonitrile, precipitate was spun down and aliquots of the clear supernatants were analyzed by RP-HPLC. For quantification of the AA oxygenase activity a solvent system consisting of acetonitrile:water:acetic acid (70:30:0.1, by vol.) was used, which baseline resolved 15-HETE and 12-HETE and allowed quantification of the 15-HETE vs 12-HETE ratio. For quantification of the resolvin E4 synthase activity a solvent system consisting of acetonitrile:water:acetic acid (50:50:0.1, by vol.) was used and under these analytical conditions resolvin E4 was eluted with a retention time of 7.6 min.

a For quantification of the relative resolvin E4 synthase activity we separately quantified the amounts of 15-HETE+12-HETE (in nmoles per incubation sample) formed during the AA oxygenase activity assays and the amounts of resolvin E4 formed in the resolvin E4 synthase activity assay. Then we set that amounts of 15-HETE+12-HETE 100% and quantified the percentage of resolvin E4 formation. The data shown indicate that the AA oxygenase activity of all enzymes tested is higher than the resolvin E4 synthase activity. In fact, for AA 15-lipoxygenating ALOX15 orthologs the resolvin E4 synthase activity amounted to about 35% of the AA oxygenase activity of these enzymes. For the AA 12-lipoxygenating ALOX15 orthologs, the resolvin E4 synthase was 6-fold lower. For each enzyme, four separate measurements of the AA oxygenase and the resolvin E4 synthase activity at two different enzyme concentrations were carried out and means ± SD are given in bold.

b *t*-test: *p* < 0.0001.

## Discussion

### Biological Relevance and Degree of Novelty

Comparing the available amino acid sequences of mammalian ALOX15 orthologs we previously hypothesized (Adel et al., 2016a; Kuhn et al., 2018) that the reaction specificity of these enzymes can be predicted on the basis of their amino acid sequences (Triad Concept). Moreover, we also suggested (Adel et al., 2016a; Kuhn et al., 2018) that mammals that are ranked in evolution below gibbons, express arachidonic acid 12-lipoxygenating ALOX15 orthologs but higher primates including humans possess 15-lipoxygenating enzymes (Evolutionary Hypothesis). The two hypotheses have predictive value but their experimental basis was somewhat limited. The experimental data reported here put the two hypotheses on a much broader and more reliable basis and we provide the following novel information:

1) Extant *Prototheria* lack ALOX15 genes: In a number of previous studies (Adel et al., 2016a; Kuhn et al., 2018; Schäfer et al., 2020; Reisch et al., 2021) functional data have been provided for some 30 mammalian ALOX15 orthologs but most of the analyzed species were classified as *Euarchontoglires*. Little information was available for *Laurasiatheria*, *Xenarthra*, *Afrotheria* and *Metatheria* and nothing was known on AA metabolizing enzymes of *Prototheria*. Here we report that the genomes of extant *Prototheria* involve different ALOX genes but that they lack ALOX15 genes (Table 1).

2) Functional characterization of novel mammalian ALOX15 orthologs: The Triad Concept and the Evolutionary Hypothesis were based on sequence data of some 60 mammalian ALOX15 orthologs and for about half of them functional data were available (Adel et al., 2016a). In the present study, we compared the ALOX15 amino acid sequences of 152 mammals representing all major mammalian subclades (Supplementary Table S1), expressed 44 novel ALOX15 orthologs (Table 2) and performed extensive mutagenesis studies of the triad determinants (Supplementary Table S1). All together, we included novel functional data for 159 mammalian ALOX15 mutants and all of them support the Triad Concept.

3) Exceptions from the Evolutionary Hypothesis: When we started this project, there were two mammalian ALOX15 orthologs (rabbit, Ord’s kangaroo rat) that did not follow the Evolutionary Hypothesis (Adel et al., 2016a; Kozlov et al., 2019). Here we report that among the 152 mammalian ALOX15 orthologs included in this study 5 (3.3%) did not follow the Evolutionary Hypothesis (Table 2). However, all of the functionally characterized ALOX15 orthologs (74 different enzymes, Supplementary Table S1) followed the Triad Concept and almost two third of them are novel.

4) Seven novel gibbon ALOX15 orthologs were functionally characterized: When the Evolutionary Hypothesis was introduced (Adel et al., 2016a) *Hylobatidea* were suggested to represent the transition taxon between arachidonic acid 12- and arachidonic acid 15-lipoxygenating ALOX15 orthologs. Unfortunately, this hypothesis was based solely on the functional characterization of the ALOX15 ortholog of *N. leucogenys* (Northern white cheeked gibbon) (Adel et al., 2016a). Here we functionally characterized the ALOX15 orthologs of seven additional gibbon species (Table 2) and the obtained data support the hypothesis that the switch in reaction specificity of mammalian ALOX15 orthologs was a targeted process and that *Hylobatidea* represents the transition taxon.

5) AA 15-lipoxygenating ALOX15 orthologs exhibit improved biosynthetic capacity for resolvin E4: In a previous study (Adel et al., 2016a) it was suggested that the evolutionary driving force for the targeted change in reaction specificity of mammalian ALOX15 orthologs was the improved biosynthetic capacity of the AA 15-lipoxygenating ALOX15 orthologs for anti-inflammatory lipoxins. Here we report that AA 15-lipoxygenating enzymes also exhibit a higher biosynthetic capacity for resolvin E4 (Table 3). Thus, the evolutionary switch ALOX15 specificity improves the biosynthetic capacity of mammalian ALOX15 orthologs for pro-resolving mediators optimizing the immune response of higher primates.

6) More than 20 novel ALOX15 cDNA sequences: For 23 of the selected mammals we could not find completely annotated ALOX15 cDNA sequences in the databases. In these cases, we downloaded all genomic sequence reads available for the corresponding species and assembled them to create searchable individual genomic databases. Finally, we screened these individual genomic databases using the human ALOX15 gene, extracted the complete cDNAs and deposited them in the GenBank Third Party Annotation database (https://www.ncbi.nlm.nih.gov/bioproject/).

Taken together, the data reported here does not only confirm the Triad Concept and the Evolutionary Hypothesis of ALOX15 specificity but also provide a large body of novel structural and functional data on mammalian ALOX15 orthologs and their evolution. Together, this data puts the two hypotheses on a much broader and more reliable experimental basis.

### ALOX15 Genes are Absent in the Genomes of Currently Sequenced *Prototheria*

Prototheria *are* the most ancient mammals and extant *Monotremata* as well as the extinct *Morganucodont*a, *Docodonta* and *Tricodonta* have been assigned to this taxon. Only some egg laying monotremes survived evolution and all of the extant representatives are indigenous to Australia and New Guinea. Unlike other mammals, monotremes possess five pairs of sex chromosomes and one of the X chromosomes resembles the Z chromosome of birds. This finding suggests that the two sex chromosomes present in all marsupial and placental mammals evolved after they split from the monotreme lineage (Veyrunes et al., 2008; Cortez et al., 2014)**.** The genome of platypus has completely been sequenced at high quality (Warren et al., 2008) and when we searched the genomic sequences for ALOX genes, we identified five different ALOX genes (Table 1). However, we could not find a functional ALOX15 gene. Similar results were obtained when we searched the genome of the short-beaked echidna (Table 1)*.* The observation that these two prototherian species lack functional ALOX15 genes but that such genes were identified in both Australidelphia and Ameridelphia suggest that these genes might have been introduced during Prototheria-Metatheria transition. This hypothesis is supported by our failure to identify functional ALOX15 genes in most representatives of non-mammalian vertebrates such as birds and fish. It should, however, been stressed that even in the genomes of some completely sequenced higher mammals such as the naked mole rat (Heterocephalus glaber) and the guinea pig (Cavia porcellus) we did not detect functional ALOX15 genes. This finding opens the possibility that eventually other Prototheria might involve functional ALOX15 genes*.*

### The Triad Concept is Applicable to all Mammalian ALOX15 Orthologs

The Triad Concept, which was developed on the basis of mutagenesis studies of selected ALOX15 orthologs (Sloane et al., 1991; Suzuki et al., 1994; Sloane et al., 1995; Borngräber et al., 1996; Berger et al., 1998; Borngräber et al., 1999; Vogel et al., 2010; Pekárová et al., 2015; Adel et al., 2016a; Kozlov et al., 2019; Schäfer et al., 2020; Reisch et al., 2021)**,** is a mechanistic tool that can be used to predict the reaction specificity of mammalian ALOX15 orthologs on the basis of their primary structures without having functional data. It is partially applicable for ALOX12 orthologs but it fails to explain the reaction specificity of ALOX15B (Vogel et al., 2010). Combined mutagenesis of the triad determinants of human (Schwarz et al., 2001), mouse (Adel et al., 2014) and zebrafish (Adel et al., 2016b) ALOX5 converted the AA 5-lipoxygenating wildtype enzymes to 15-lipoxygenating catalysts and thus, this concept might also be applicable for ALOX5 orthologs. In principle, the Triad Concept suggests that Phe(F)353, Ile(I)418 + Met(M)419, Ile(I)593 (amino acid numbering for human ALOX15) form the bottom of the boot-shaped substrate binding pocket and that the side chain geometry of these residues determines the depth of this cavity (Borngräber et al., 1999; Ivanov et al., 2010). If small amino acids are localized at these positions, the substrate fatty acids may penetrate deeply into the pocket, which favors AA 12-lipoxygenation. In contrast, when more space-filling residues are located at these positions AA 15-lipoxygenation is preferred (Scheme 1).

However, the triad determinants are not equally important and the following ranking may be suggested: 1) If a Leu(L) or a smaller amino acid is located at position 353 [Leu(L)353] the enzyme is AA 12-lipoxygenating regardless of which amino acids are located at the other triad positions. 2) If a bulky Phe(F) (Phe(F)353) is located at this position, as it is the case for the vast majority of mammalian ALOX15 orthologs (Supplementary Tables S1, S2), the reaction specificity of the enzyme depends on the side-chain geometry of the amino acids located at positions 418 + 419. If space-filling residues such as Ile(I)+Met(M) [Ile(I)418 + Met(M)419] are located at these positions AA 15-lipoxygenation is catalyzed. In contrast, when smaller residues are located at these positions [Val(V)418 + Val(V)419] AA 12-lipoxygenation is preferred. 3) Ile(I)593 and Thr(T)594 modify the reaction specificity but may not play a decisive role for most enzymes. To put the Triad Concept on a broader experimental basis we first explored whether the ALOX15 orthologs carrying a Leu(L) at position 353 (Borngraber-1 determinant, BG1) catalyze AA 12-lipoxygenation. Here we found that all functionally characterized ALOX15 orthologs of this enzyme type [rabbit (12), house mouse, brown rat, black rat, African woodland thicket rat] catalyze AA 12-lipoxygenation regardless of the chemical identity of the other triad determinants. When the Leu(L)/Ile(I)353 residues in these enzymes were mutated to a more space-filling Phe(F) AA 15-lipoxygenation was dominant (Supplementary Table S2) and these data confirm the functional importance of this amino acid. Next, we explored the role of Ile(I)418 + Met(M)419 (Sloane determinants, SL). For all functionally characterized ALOX15 orthologs carrying the Phe(F)353 + Ile(I) (Leu(L))418 + Met(M)419 motif (extant and extinct human subspecies, chimpanzee, bonobo, gorilla, orangutan, most gibbon species, rabbit (15), mountain hare, Ord’s kangaroo rat, collared anteater, giant anteater) dominant AA 15-lipoxygenation was observed. When smaller amino acids were introduced at Ile(I)418 and/or Met(M)419 (Ile(I)418Ala(A), Met(M)419Ala(A), Ile(I)418Val(V)+Met(M)419Val(V)) AA 12-lipoxygenating mutants resulted (Supplementary Table S2). Finally, we tested experimentally whether the Triad Concept is also applicable to the ALOX15 orthologs carrying the Phe(F)353 + Val(V)418 + Val(V)419 motif. Among mammalian ALOX15 orthologs, these enzymes are dominant since the overwhelming majority of *Euarchontoglires*, all *Laurasiatheria*, all *Afrotheria* and all *Metatheria* express such ALOX15 orthologs. In these enzymes, we first introduced more bulky residues at Val(V)418 or Val(V)419 and observed increased 15-HETE formation. For the corresponding double mutants 15-HETE formation was always dominant (Supplementary Table S2). In most cases, these alterations could be reversed when a small residue (Leu(L), Ala(A)) was consecutively introduced at Phe(F)353.

In summary, one can conclude that the ALOX15 orthologs of more than 70 different mammals have functionally been characterized and that extensive mutagenesis studies of the triad determinants suggest that all enzymes follow the Triad Concept. We did not find a single exception.

### The Vast Majority of Mammalian ALOX15 Orthologs Follow the Evolutionary Hypothesis of ALOX15 Specificity

**SCHEME 2:**
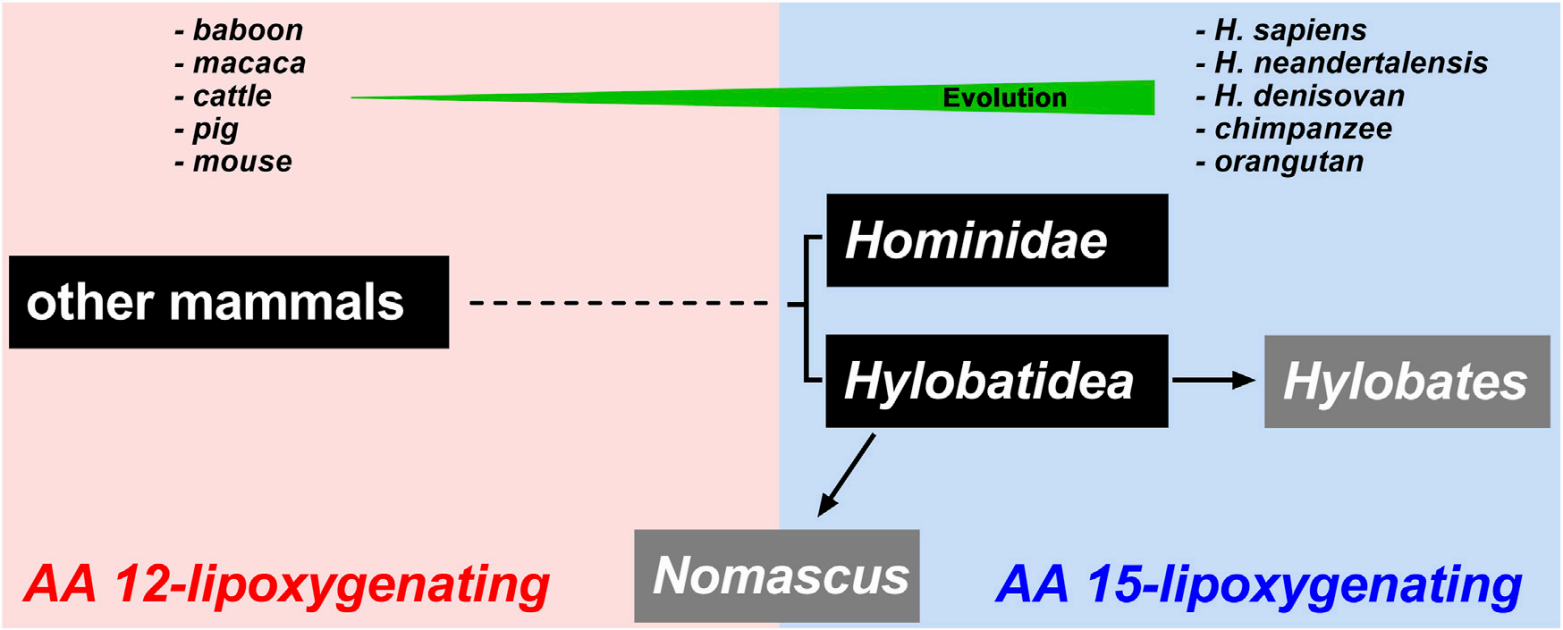
Schematic representation of the Evolutionary Hypothesis of ALOX15 reaction specificity. Lower mammals including mouse, cattle and baboon express AA 12-lipoxygenating ALOX15 orthologs. Higher primates including extant and extinct human subspecies, chimpanzee and orangutan express AA 15-lipoxygenating ALOX15 orthologs. *Hylobatidea* represents a transition taxon that involves species expressing AA 15-lipoxygenating orthologs (*Hylobates*) but also species expressing ALOX15 orthologs with pronounced dual reaction specificity (*Nomascus*). In fact, the ALOX15 orthologs of different *Nomascus* species oxygenate arachidonic acid to 12-HETE and 15-HETE in similar amounts.

Mammalian ALOX15 orthologs have been classified as 15-lipoxygenases since the human ortholog catalyzed dominant AA 15-lipoxygenation. However, the vast majority of these enzymes (Supplementary Table S1) including the orthologs of pigs (Yokoyama et al., 1986), cattle (De Marzo et al., 1992), rats (Watanabe et al., 1993), and mice (Freire-Moar et al., 1995) catalyze AA 12-lipoxygenation. In other words, in mammals, AA 15-lipoxygenating and AA 12-lipoxygenating ALOX15 orthologs coexist but the AA 12-lipoxygenating enzymes are dominant. There is, however not a random distribution of AA12-and AA15-lipoxygenating ALOX15 orthologs. In fact, mammals ranked in evolution above gibbons, such as extant and extinct human subspecies, bonobo, chimpanzee, gorilla and orangutans express AA 15-lipoxygenating ALOX15 orthologs (Adel et al., 2016a). In contrast, mammals ranked below gibbons express AA 12-lipoxygenating ALOX15 orthologs (Scheme 2). Gibbons were considered a transition clade since the ALOX15 ortholog of *Nomascus leucogenys* converted AA to almost equal amounts of 12- and 15-HETE (Adel et al., 2016a). Although this Evolutionary Hypothesis of ALOX15 specificity was supported by more recent studies (Kozlov et al., 2019; Schäfer et al., 2020; Reisch et al., 2021) there were a number of exceptions.

For instance, rabbits, which are clearly ranked below gibbons in evolution, express both an AA 12- and an AA 15-lipoxygenating ALOX15 (Berger et al., 1998). The two isoforms originate from the same gene, which encodes for the Phe(F)353-Ile(I)418 + Met(M)419 triad motif. It still remains unclear why in rabbit peripheral monocytes an AA 12-lipoxygenating isoform involving the Leu(L)353-Val(V)148 + Val(V)419 motif is expressed. Another exception from the Evolutionary Hypothesis is the ALOX15 ortholog of the Ord’s kangaroo rat (Kozlov et al., 2019). These animals are ranked below gibbons but its ALOX15 gene encodes for an AA 15-lipoxygenating ALOX15 ortholog (Supplementary Table S1). In this study, we found four additional exceptions: 1) As rabbits mountain hares are classified as *Lagomorpha* (Figure 4) and the ALOX15 genes of these two mammalian species carry the Phe(F)353-Ile(I)418 + Met(M)419 motif. These sequence data suggest AA 15-lipoxygenating ALOX15 orthologs and *in vitro* activity assays confirmed this prediction (Supplementary Table S1). However, since the ALOX15 ortholog of the American pika, which also belongs to *Lagomorpha*, is AA 12-lipoxgenating it can be concluded that not all *Lagomorpha* violate the Evolutionary Hypothesis of ALOX15 specificity. 2) The ALOX15 ortholog of the hoary bamboo rat, which carries the Phe(F)353-Val(V)418 + Met(M)419 motif, functions as AA 15-lipoxygenating enzyme. Although this enzyme follows the Triad Concept (Supplementary Table S2) it violates the Evolutionary Hypothesis. Bamboo rats are ranked below gibbons but expresses an AA 15-lipoxygenating ALOX15 ortholog. 3) The ALOX15 ortholog of the meadow jumping mouse, which also carries the Phe353-Ile418 + Val419 motif (Supplementary Table S1), oxygenated arachidonic acid to a 6:4 mixture of 15- and 12-HETE. Here again, the enzyme follows the Triad Concept (Supplementary Table S2) but violates the Evolutionary Hypothesis. 4) The ALOX15 orthologs of the collared anteater and the giant anteater, which are classified as *Xenarthra*, also carry the Phe(F)353 + Leu(L)418 + Met(M)419 motif. Based on the Triad Concept AA 15-lipoxygenation was predicted and *in vitro* activity assays confirmed this conclusion (Supplementary Table S1). Thus, these two ALOX15 orthologs also violate the Evolutionary Hypothesis. On the other hand, the ALOX15 orthologs of the screaming hairy armadillo and the nine-banded armadillo, which are also classified as *Xenarthra*, follow the Evolutionary Hypothesis.

In summary, one can conclude that the vast majority of mammalian ALOX15 orthologs follow the Evolutionary Hypothesis of ALOX15 specificity. All together, we extracted 152 ALOX15 sequences from the databases and predicted the reaction specificity of the corresponding enzymes based on the Triad Concept. We found that 145 species follow the Evolutionary Hypothesis but 7 (4.6%) violated it. When we performed this calculation with those ALOX15 orthologs, for which functional data are currently available, we found that from the 74 ALOX15 orthologs, which have been functionally characterized, seven enzymes (9.3%) did not follow the Evolutionary Hypothesis. It should, however, been stressed that for functional characterization we focused on those ALOX15 orthologs carrying unusual triad determinants. Enzymes carrying the canonical motif [Phe(F)353-Val(V)418 + Val(V)419] for AA 12-lipoxygenating ALOX15 orthologs were frequently not characterized. Since these enzymes are likely to exhibit AA 12-lipoxygenating activities it might be concluded that more than 95% of all mammalian ALOX15 orthologs follow the Evolutionary Hypothesis of ALOX15 specificity.

### The Driving Forces for the Evolutionary Switch in Reaction Specificity of ALOX15 Orthologs

In a previous study it was reported that mammalian AA 15-lipoxygenating ALOX15 orthologs exhibit an elevated lipoxin synthase activity when 5-HETE or 5,6-diHETE were employed as substrates (Adel et al., 2016a). Since lipoxins have been characterized as anti-inflammatory and pro-resolving signaling molecules (Ryan and Godson, 2010; Romano, 2010) it was hypothesized that mammals expressing AA 15-lipoxygenating ALOX15 orthologs can more efficiently control the inflammatory reaction (Adel et al., 2016a). If this hypothesis is true, the evolutionary switch in the reaction specificity of mammalian ALOX15 orthologs might be considered as part of a more general concept aimed at optimizing the immune system during late primate evolution. Resolvin E4 is a similar pro-resolving mediator, which can be synthesized *via* the Alox15 pathway. For this study, we compared the resolvin E4 synthase activity of selected mammalian AA 12-lipoxygenating and AA 15-lipoxygenating ALOX15 isoforms using 5*S*-HEPE as a substrate and observed a 6-fold higher resolvin E4 synthase activity for the AA-15-lipoxygenating enzymes (Table 3). This finding is consistent with the increased lipoxin synthase activity of these ALOX15 orthologs reported previously (Adel et al., 2016a). There are, however, a number of other potential scenarios. In theory, AA 15-lipoxygenating ALOX15 isoforms should exhibit a higher linoleic acid oxygenase activity than their AA 12-lipoxygenating counterparts since this fatty acid lacks a n-9 bisallylic methylene, which is required for AA 12-lipoxygenation. Thus, AA 15-lipoxygenating ALOX15 orthologs might exhibit a broader substrate specificity. Moreover, since linoleic acid is a major constituent of biomembranes it may be predicted, that the membrane oxygenase activity of AA 15-lipoxygenating ALOX15 orthologs might also be higher than that of AA 12-lipoxygenating enzymes. Consequently, more work is needed to clarify the evolutionary driving force for the switch in reaction specificity of mammalian ALOX15 orthologs.

## Data Availability

The datasets presented in this study can be found in online repositories. The names of the repository/repositories and accession number(s) can be found in the article/Supplementary Material.

## References

[B1] Adel S. Heydeck D. Kuhn H. Ufer C. (2016). The Lipoxygenase Pathway in Zebrafish. Expression and Characterization of Zebrafish ALOX5 and Comparison with its Human Ortholog. Biochim. Biophys. Acta (Bba) - Mol. Cel Biol. Lipids 1861 (1), 1–11. 10.1016/j.bbalip.2015.10.001 26456699

[B2] Adel S. Hofheinz K. Heydeck D. Kuhn H. Häfner A.-K. (2014). Phosphorylation Mimicking Mutations of ALOX5 Orthologs of Different Vertebrates Do Not Alter Reaction Specificities of the Enzymes. Biochim. Biophys. Acta (Bba) - Mol. Cel Biol. Lipids 1841 (10), 1460–1466. 10.1016/j.bbalip.2014.07.005 25025884

[B3] Adel S. Kakularam K. R. Horn T. Reddanna P. Kuhn H. Heydeck D. (2015). Leukotriene Signaling in the Extinct Human Subspecies Homo Denisovan and Homo Neanderthalensis. Structural and Functional Comparison with *Homo sapiens* . Arch. Biochem. Biophys. 565, 17–24. 10.1016/j.abb.2014.10.012 25447821

[B4] Adel S. Karst F. González-Lafont À. Pekárová M. Saura P. Masgrau L. (2016). Evolutionary Alteration of ALOX15 Specificity Optimizes the Biosynthesis of Antiinflammatory and Proresolving Lipoxins. Proc. Natl. Acad. Sci. U.S.A. 113 (30), E4266–E4275. 10.1073/pnas.1604029113 27412860 PMC4968705

[B5] Aldrovandi M. Banthiya S. Meckelmann S. Zhou Y. Heydeck D. O'Donnell V. B. (2018). Specific Oxygenation of Plasma Membrane Phospholipids by *Pseudomonas aeruginosa* Lipoxygenase Induces Structural and Functional Alterations in Mammalian Cells. Biochim. Biophys. Acta (Bba) - Mol. Cel Biol. Lipids 1863, 152–164. 10.1016/j.bbalip.2017.11.005 PMC5764228 29146531

[B6] Belkner J. Stender H. Kühn H. (1998). The Rabbit 15-lipoxygenase Preferentially Oxygenates LDL Cholesterol Esters, and This Reaction Does Not Require Vitamin E. J. Biol. Chem. 273 (36), 23225–23232. 10.1074/jbc.273.36.23225 9722553

[B7] Berger M. Schwarz K. Thiele H. Reimann I. Huth A. Borngräber S. (1998). Simultaneous Expression of Leukocyte-type 12-lipoxygenase and Reticulocyte-type 15-lipoxygenase in Rabbits 1 1Edited by F. Cohen. J. Mol. Biol. 278 (5), 935–948. 10.1006/jmbi.1998.1737 9600854

[B8] Blanchette M. Green E. D. Miller W. Haussler D. (2004). Reconstructing Large Regions of an Ancestral Mammalian Genome In Silico. Genome Res. 14 (12), 2412–2423. 10.1101/gr.2800104 15574820 PMC534665

[B9] Boeglin W. E. Kim R. B. Brash A. R. (1998). A 12 R -lipoxygenase in Human Skin: Mechanistic Evidence, Molecular Cloning, and Expression. Proc. Natl. Acad. Sci. U.S.A. 95 (12), 6744–6749. 10.1073/pnas.95.12.6744 9618483 PMC22619

[B10] Borngräber S. Browner M. Gillmor S. Gerth C. Anton M. Fletterick R. (1999). Shape and Specificity in Mammalian 15-Lipoxygenase Active Site. J. Biol. Chem. 274 (52), 37345–37350. 10.1074/jbc.274.52.37345 10601303

[B11] Borngräber S. Kuban R.-J. Anton M. Kühn H. (1996). Phenylalanine 353 Is a Primary Determinant for the Positional Specificity of Mammalian 15-lipoxygenases. J. Mol. Biol. 264 (5), 1145–1153. 10.1006/jmbi.1996.0702 9000636

[B12] Brash A. R. Boeglin W. E. Chang M. S. (1997). Discovery of a Second 15 S -lipoxygenase in Humans. Proc. Natl. Acad. Sci. U.S.A. 94 (12), 6148–6152. 10.1073/pnas.94.12.6148 9177185 PMC21017

[B13] Bryant R. W. Bailey J. M. Schewe T. Rapoport S. M. (1982). Positional Specificity of a Reticulocyte Lipoxygenase. Conversion of Arachidonic Acid to 15-S-Hydroperoxy-Eicosatetraenoic Acid. J. Biol. Chem. 257 (11), 6050–6055. 10.1016/s0021-9258(20)65103-1 6804460

[B14] Budd G. E. Jensen S. (2000). A Critical Reappraisal of the Fossil Record of the Bilaterian Phyla. Biol. Rev. 75 (2), 253–295. 10.1017/s000632310000548x 10881389

[B15] Burgin C. J. Colella J. P. Kahn P. L. Upham N. S. (2018). How many Species of Mammals Are There? J. Mammal 99 (1), 1–14. 10.1093/jmammal/gyx147

[B16] Chaitidis P. Adel S. Anton M. Heydeck D. Kuhn H. Horn T. (2013). Lipoxygenase Pathways in Homo Neanderthalensis: Functional Comparison with *Homo sapiens* Isoforms. J. lipid Res. 54 (5), 1397–1409. 10.1194/jlr.M035626 23475662 PMC3622333

[B17] Chen X. S. Kurre U. Jenkins N. A. Copeland N. G. Funk C. D. (1994). cDNA Cloning, Expression, Mutagenesis of C-Terminal Isoleucine, Genomic Structure, and Chromosomal Localizations of Murine 12-lipoxygenases. J. Biol. Chem. 269 (19), 13979–13987. 10.1016/s0021-9258(17)36743-1 8188678

[B18] Cortez D. Marin R. Toledo-Flores D. Froidevaux L. Liechti A. Waters P. D. (2014). Origins and Functional Evolution of Y Chromosomes across Mammals. Nature 508 (7497), 488–493. 10.1038/nature13151 24759410

[B19] Cyrus T. Witztum J. L. Rader D. J. Tangirala R. Fazio S. Linton M. F. (1999). Disruption of the 12/15-lipoxygenase Gene Diminishes Atherosclerosis in Apo E-Deficient Mice. J. Clin. Invest. 103 (11), 1597–1604. 10.1172/JCI5897 10359569 PMC408369

[B20] De Marzo N. Sloane D. L. Dicharry S. Highland E. Sigal E. (1992). Cloning and Expression of an Airway Epithelial 12-lipoxygenase. Am. J. Physiol. 263 (2 Pt 1), 1–L1. 10.1152/ajplung.1992.262.2.L198 1636733

[B21] Freire M. O. Van Dyke T. E. (2000). Natural Resolution of Inflammation. Periodontol. 2000 63 (1), 149–164. 10.1111/prd.12034 PMC4022040 23931059

[B22] Freire-Moar J. Alavi-Nassab A. Ng M. Mulkins M. Sigal E. (1995). Cloning and Characterization of a Murine Macrophage Lipoxygenase. Biochim. Biophys. Acta (Bba) - Lipids Lipid Metab. 1254 (1), 112–116. 10.1016/0005-2760(94)00199-9 7811740

[B23] Funk C. D. Chen X. S. Johnson E. N. Zhao L. (2002). Lipoxygenase Genes and Their Targeted Disruption. Prostaglandins Other Lipid Mediat 68-69, 303–12. 10.1016/s0090-6980(02)00036-9 12432925

[B24] Groves C. P. (2001). *Primate Taxonomy* Smithsonian. Washington, DC: Institution Press, 350.

[B25] Haeggström J. Z. Funk C. D. (2011). Lipoxygenase and Leukotriene Pathways: Biochemistry, Biology, and Roles in Disease. Chem. Rev. 111 (10), 5866–5898. 10.1021/cr200246d 21936577

[B26] Hamberg M. Samuelsson B. (1974). Prostaglandin Endoperoxides. Novel Transformations of Arachidonic Acid in Human Platelets. Proc. Natl. Acad. Sci. U.S.A. 71 (9), 3400–3404. 10.1073/pnas.71.9.3400 4215079 PMC433780

[B27] Ivanov I. Heydeck D. Hofheinz K. Roffeis J. O’Donnell V. B. Kuhn H. (2010). Molecular Enzymology of Lipoxygenases. Arch. Biochem. Biophys. 503 (2), 161–174. 10.1016/j.abb.2010.08.016 20801095

[B28] Ivanov I. Kuhn H. Heydeck D. (2015). Structural and Functional Biology of Arachidonic Acid 15-lipoxygenase-1 (ALOX15). Gene 573 (1), 1–32. 10.1016/j.gene.2015.07.073 26216303 PMC6728142

[B29] Johannesson M. Backman L. Claesson H.-E. Forsell P. K. A. (2010). Cloning, Purification and Characterization of Non-human Primate 12/15-lipoxygenases. Prostaglandins, Leukot. Essent. Fatty Acids 82 (2-3), 121–129. 10.1016/j.plefa.2009.11.006 20106647

[B30] Kozlov N. Humeniuk L. Ufer C. Ivanov I. Golovanov A. Stehling S. (2019). Functional Characterization of Novel ALOX15 Orthologs Representing Key Steps in Mammalian Evolution Supports the Evolutionary Hypothesis of Reaction Specificity. Biochim. Biophys. Acta (Bba) - Mol. Cel Biol. Lipids 1864 (3), 372–385. 10.1016/j.bbalip.2018.12.016 30599203

[B31] Kuhn H. Banthiya S. van Leyen K. (2015). Mammalian Lipoxygenases and Their Biological Relevance. Biochim. Biophys. Acta (Bba) - Mol. Cel Biol. Lipids 1851 (4), 308–330. 10.1016/j.bbalip.2014.10.002 PMC4370320 25316652

[B32] Kühn H. Barnett J. Grunberger D. Baecker P. Chow J. Nguyen B. (1993). Overexpression, Purification and Characterization of Human Recombinant 15-lipoxygenase. Biochim. Biophys. Acta (Bba) - Lipids Lipid Metab. 1169 (1), 80–89. 10.1016/0005-2760(93)90085-n 8334154

[B33] Kühn H. Belkner J. Suzuki H. Yamamoto S. (1994). Oxidative Modification of Human Lipoproteins by Lipoxygenases of Different Positional Specificities. J. lipid Res. 35 (10), 1749–1759. 10.1016/s0022-2275(20)39770-4 7852852

[B34] Kuhn H. Belkner J. Wiesner R. Brash A. R. (1990). Oxygenation of Biological Membranes by the Pure Reticulocyte Lipoxygenase. J. Biol. Chem. 265 (30), 18351–18361. 10.1016/s0021-9258(17)44759-4 2120232

[B35] Kühn H. Heydeck D. Hugou I. Gniwotta C. (1997). *In Vivo* action of 15-lipoxygenase in Early Stages of Human Atherogenesis. J. Clin. Invest. 99 (5), 888–893. 10.1172/JCI119253 9062346 PMC507896

[B36] Kuhn H. Humeniuk L. Kozlov N. Roigas S. Adel S. Heydeck D. (2018). The Evolutionary Hypothesis of Reaction Specificity of Mammalian ALOX15 Orthologs. Prog. lipid Res. 72, 55–74. 10.1016/j.plipres.2018.09.002 30237084

[B37] Kumar V. Hallström B. M. Janke A. (2013). Coalescent-based Genome Analyses Resolve the Early Branches of the Euarchontoglires. PloS one 8 (4), e60019. 10.1371/journal.pone.0060019 23560065 PMC3613385

[B38] Mashima R. Okuyama T. (2015). The Role of Lipoxygenases in Pathophysiology; New Insights and Future Perspectives. Redox Biol. 6, 297–310. 10.1016/j.redox.2015.08.006 26298204 PMC4556770

[B39] Nilsson M. A. Churakov G. Sommer M. Tran N. V. Zemann A. Brosius J. (2010). Tracking Marsupial Evolution Using Archaic Genomic Retroposon Insertions. Plos Biol. 8 (7), e1000436. 10.1371/journal.pbio.1000436 20668664 PMC2910653

[B40] O'Leary M. A. Bloch J. I. Flynn J. J. Gaudin T. J. Giallombardo A. Giannini N. P. (2013). The Placental Mammal Ancestor and the post-K-Pg Radiation of Placentals. Science 339 (6120), 662–667. 10.1126/science.1229237 23393258

[B41] O’Leary M. A. Bloch J. I. Flynn J. J. Gaudin T. J. Giallombardo A. Giannini N. P. (2013). Response to Comment on "The Placental Mammal Ancestor and the Post-K-Pg Radiation of Placentals". Science 341 (6146), 613. 10.1126/science.1238162 23929968

[B42] Pekárová M. Kuhn H. Bezáková L. Ufer C. Heydeck D. (2015). Mutagenesis of Triad Determinants of Rat Alox15 Alters the Specificity of Fatty Acid and Phospholipid Oxygenation. Arch. Biochem. Biophys. 571, 50–57. 10.1016/j.abb.2015.02.029 25731857

[B43] Rådmark O. Werz O. Steinhilber D. Samuelsson B. (2015). 5-Lipoxygenase, a Key Enzyme for Leukotriene Biosynthesis in Health and Disease. Biochim. Biophys. Acta (Bba) - Mol. Cel Biol. Lipids 1851 (4), 331–339. 10.1016/j.bbalip.2014.08.012 25152163

[B44] Rapoport S. M. Schewe T. (1986). The Maturational Breakdown of Mitochondria in Reticulocytes. Biochim. Biophys. Acta 864 (3-4), 471–495. 10.1016/0304-4157(86)90006-7 3098292

[B45] Rauhut O. W. M. Martin T. Ortiz-Jaureguizar E. Puerta P. (2002). A Jurassic Mammal from South America. Nature 416 (6877), 165–168. 10.1038/416165a 11894091

[B46] Reilly S. M. White T. D. (2003). Hypaxial Motor Patterns and the Function of Epipubic Bones in Primitive Mammals. Science 299 (5605), 400–402. 10.1126/science.1074905 12532019

[B47] Reisch F. Kakularam K. R. Stehling S. Heydeck D. Kuhn H. (2021). Eicosanoid Biosynthesis in marine Mammals. Febs J. 288 (4), 1387–1406. 10.1111/febs.15469 32627384

[B48] Romano M. (2010). Lipoxin and Aspirin-Triggered Lipoxins. The Scientific World JOURNAL 10, 1048–1064. 10.1100/tsw.2010.113 20526535 PMC5763664

[B49] Ryan A. Godson C. (2010). Lipoxins: Regulators of Resolution. Curr. Opin. Pharmacol. 10 (2), 166–172. 10.1016/j.coph.2010.02.005 20226737

[B50] Schäfer M. Fan Y. Gu T. Heydeck D. Stehling S. Ivanov I. (2020). The Lipoxygenase Pathway of Tupaia Belangeri Representing Scandentia. Genomic Multiplicity and Functional Characterization of the ALOX15 Orthologs in the Tree Shrew. Biochim. Biophys. Acta (Bba) - Mol. Cel Biol. Lipids 1865 (2), 158550. 10.1016/j.bbalip.2019.158550 31676437

[B51] Schewe T. Halangk W. Hiebsch C. Rapoport S. M. (1975). A Lipoxygenase in Rabbit Reticulocytes Which Attacks Phospholipids and Intact Mitochondria. FEBS Lett. 60 (1), 149–152. 10.1016/0014-5793(75)80439-x 6318

[B52] Schwarz K. Walther M. Anton M. Gerth C. Feussner I. Kuhn H. (2001). Structural Basis for Lipoxygenase Specificity. J. Biol. Chem. 276 (1), 773–779. 10.1074/jbc.M005114200 11027682

[B53] Seiffert E. R. (2007). A New Estimate of Afrotherian Phylogeny Based on Simultaneous Analysis of Genomic, Morphological, and Fossil Evidence. BMC Evol. Biol. 7, 224. 10.1186/1471-2148-7-224 17999766 PMC2248600

[B54] Serhan C. N. Petasis N. A. (2011). Resolvins and Protectins in Inflammation Resolution. Chem. Rev. 111 (10), 5922–5943. 10.1021/cr100396c 21766791 PMC3192290

[B55] Sigal E. Grunberger D. Highland E. Gross C. Dixon R. A. Craik C. S. (1990). Expression of Cloned Human Reticulocyte 15-lipoxygenase and Immunological Evidence that 15-lipoxygenases of Different Cell Types Are Related. J. Biol. Chem. 265 (9), 5113–5120. 10.1016/s0021-9258(19)34092-x 2318885

[B56] Sloane D. L. Leung R. Barnett J. Craik C. S. Sigal E. (1995). Conversion of Human 15-lipoxygenase to an Efficient 12-lipoxygenase: the Side-Chain Geometry of Amino Acids 417 and 418 Determine Positional Specificity. Protein Eng. Des. Sel 8 (3), 275–282. 10.1093/protein/8.3.275 7479689

[B57] Sloane D. L. Leung R. Craik C. S. Sigal E. (1991). A Primary Determinant for Lipoxygenase Positional Specificity. Nature 354 (6349), 149–152. 10.1038/354149a0 1944593

[B58] Springer M. S. Meredith R. W. Teeling E. C. Murphy W. J. (2013). Technical Comment on "The Placental Mammal Ancestor and the Post-K-Pg Radiation of Placentals". Science 341 (6146), 613. 10.1126/science.1238025 23929967

[B59] Stanhope M. J. Waddell V. G. Madsen O. de Jong W. Hedges S. B. Cleven G. C. (1998). Molecular Evidence for Multiple Origins of Insectivora and for a New Order of Endemic African Insectivore Mammals. Proc. Natl. Acad. Sci. U.S.A. 95 (17), 9967–9972. 10.1073/pnas.95.17.9967 9707584 PMC21445

[B60] Suzuki H. Kishimoto K. Yoshimoto T. Yamamoto S. Kanai F. Ebina Y. (1994). Site-directed Mutagenesis Studies on the Iron-Binding Domain and the Determinant for the Substrate Oxygenation Site of Porcine Leukocyte Arachidonate 12-lipoxygenase. Biochim. Biophys. Acta (Bba) - Lipids Lipid Metab. 1210 (3), 308–316. 10.1016/0005-2760(94)90234-8 8305485

[B61] Tabuce R. Marivaux L. Adaci M. Bensalah M. Hartenberger J.-L. Mahboubi M. (2007). Early Tertiary Mammals from North Africa Reinforce the Molecular Afrotheria Clade. Proc. R. Soc. B. 274 (1614), 1159–1166. 10.1098/rspb.2006.0229 PMC2189562 17329227

[B62] van Leyen K. Duvoisin R. M. Engelhardt H. Wiedmann M. (1998). A Function for Lipoxygenase in Programmed Organelle Degradation. Nature 395 (6700), 392–395. 10.1038/26500 9759730

[B63] Veyrunes F. Waters P. D. Miethke P. Rens W. McMillan D. Alsop A. E. (2008). Bird-like Sex Chromosomes of Platypus Imply Recent Origin of Mammal Sex Chromosomes. Genome Res. 18 (6), 965–973. 10.1101/gr.7101908 18463302 PMC2413164

[B64] Vogel R. Jansen C. Roffeis J. Reddanna P. Forsell P. Claesson H.-E. (2010). Applicability of the Triad Concept for the Positional Specificity of Mammalian Lipoxygenases. J. Biol. Chem. 285 (8), 5369–5376. 10.1074/jbc.M109.057802 20026599 PMC2820765

[B65] Warren W. C. Hillier L. W. Marshall Graves J. A. Birney E. Ponting C. P. Grutzner F. (2008). Genome Analysis of the Platypus Reveals Unique Signatures of Evolution. Nature 453 (7192), 175–183. 10.1038/nature06936 18464734 PMC2803040

[B66] Watanabe T. Haeggstrom J. Z. (1993). Rat 12-Lipoxygenase: Mutations of Amino Acids Implicated in the Positional Specificity of 15- and 12-Lipoxygenase. Biochem. biophysical Res. Commun. 192 (3), 1023–1029. 10.1006/bbrc.1993.1519 8507177

[B67] Watanabe T. Medina J. F. Haeggstrom J. Z. Radmark O. Samuelsson B. (1993). Molecular Cloning of a 12-lipoxygenase cDNA from Rat Brain. Eur. J. Biochem. 212 (2), 605–612. 10.1111/j.1432-1033.1993.tb17699.x 8444196

[B68] Yokoyama C. Shinjo F. Yoshimoto T. Yamamoto S. Oates J. A. Brash A. R. (1986). Arachidonate 12-lipoxygenase Purified from Porcine Leukocytes by Immunoaffinity Chromatography and its Reactivity with Hydroperoxyeicosatetraenoic Acids. J. Biol. Chem. 261 (35), 16714–16721. 10.1016/s0021-9258(18)66623-2 3782139

[B69] Zheng Y. Brash A. R. (2010). Dioxygenase Activity of Epidermal Lipoxygenase-3 Unveiled. J. Biol. Chem. 285 (51), 39866–39875. 10.1074/jbc.M110.155374 20921226 PMC3000968

