# Supplementary material for: The Reaction Specificity of Mammalian ALOX15 Orthologs is Changed During Late Primate Evolution and These Alterations Might Offer Evolutionary Advantages for Hominidae
Source: Front Cell Dev Biol. 2022 Apr 21;10:871585. doi: 10.3389/fcell.2022.871585 (PMC9068934; doi:10.3389/fcell.2022.871585)
Supplement: Supplementary file 2 [file DataSheet1.pdf]

Supplemental information to the paper

The reaction specificity of mammalian ALOX15 orthologs is changed during late primate evolution and these alterations might offer evolutionary advantages for *Hominidae*

Dagmar Heydeck[1*], Florian Reisch[1,2], Marjann Schäfer[1,2], Kumar R. Kakularam[1], Sophie A. Roigas[1], Sabine Stehling[1], Gerhard P. Püschel[2] and Hartmut Kuhn[1]

[1]Charité – Universitätsmedizin Berlin, corporate member of Freie Universität Berlin and Humboldt Universität zu Berlin, Institute of Biochemistry, Charitéplatz 1, D-10117 Berlin, Germany.

[2]Institute for Nutritional Sciences, University Potsdam, Arthur-Scheunert-Allee 114-116, 14558 Nuthetal, Germany

Running title: evolution of ALOX15 specificity

Keywords: eicosanoids, lipid peroxidation, oxidative stress, recombinant proteins, ferroptosis,

***correspondence to:** dagmar.heydeck@charite.de

Figure S1. Simplified classification of mammals and overview of the mammalian ALOX15 orthologs that have been characterized in this study. Mammals can be classified in *Prototheria*, *Metatheria* and *Eutheria* and each of these clades can further be subclassified. For this study we extracted the sequences of the ALOX15 cDNAs of different mammalian species (given in blue) and specified the triad determinants (indicated in red) by dual amino acid alignment with human and ALOX15. The one letter code for amino acids was employed. More detailed subclassifications of *Eutheria* is given in **Fig. S3-6** and representative species with their triad determinants are given in these images.

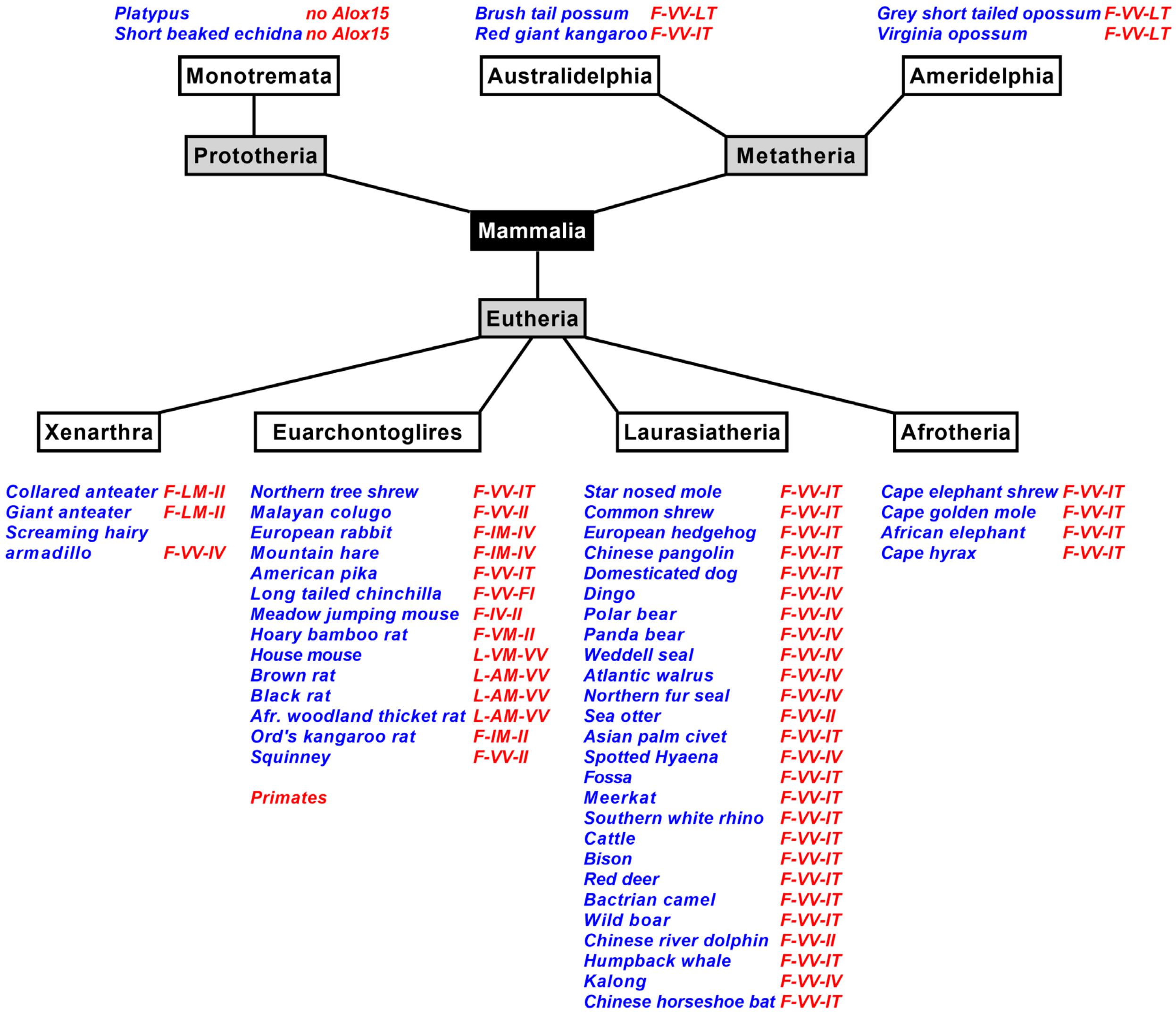

Figure S2. Reaction specificity of primate ALOX15 orthologs. Primates can be subclassified in two suborders (*Haplorrhini*, *Strepsirrhini*). ALOX15 sequences sequences of randomly selected representatives of these two primate suborders (given in blue) were extracted and their triad determinants are given in red. ALOX15 orthologs with AA 12-lipoxygenating activity are indicated by the light brown background. Enzyme orthologs on dark brown background represent AA 15-lipoxygenating ALOX15 orthologs. *Nomascus* species (yellow cheeked gibbon, northern white-cheeked gibbon) express ALOX15 orthologs with pronounced dual reaction specificity.

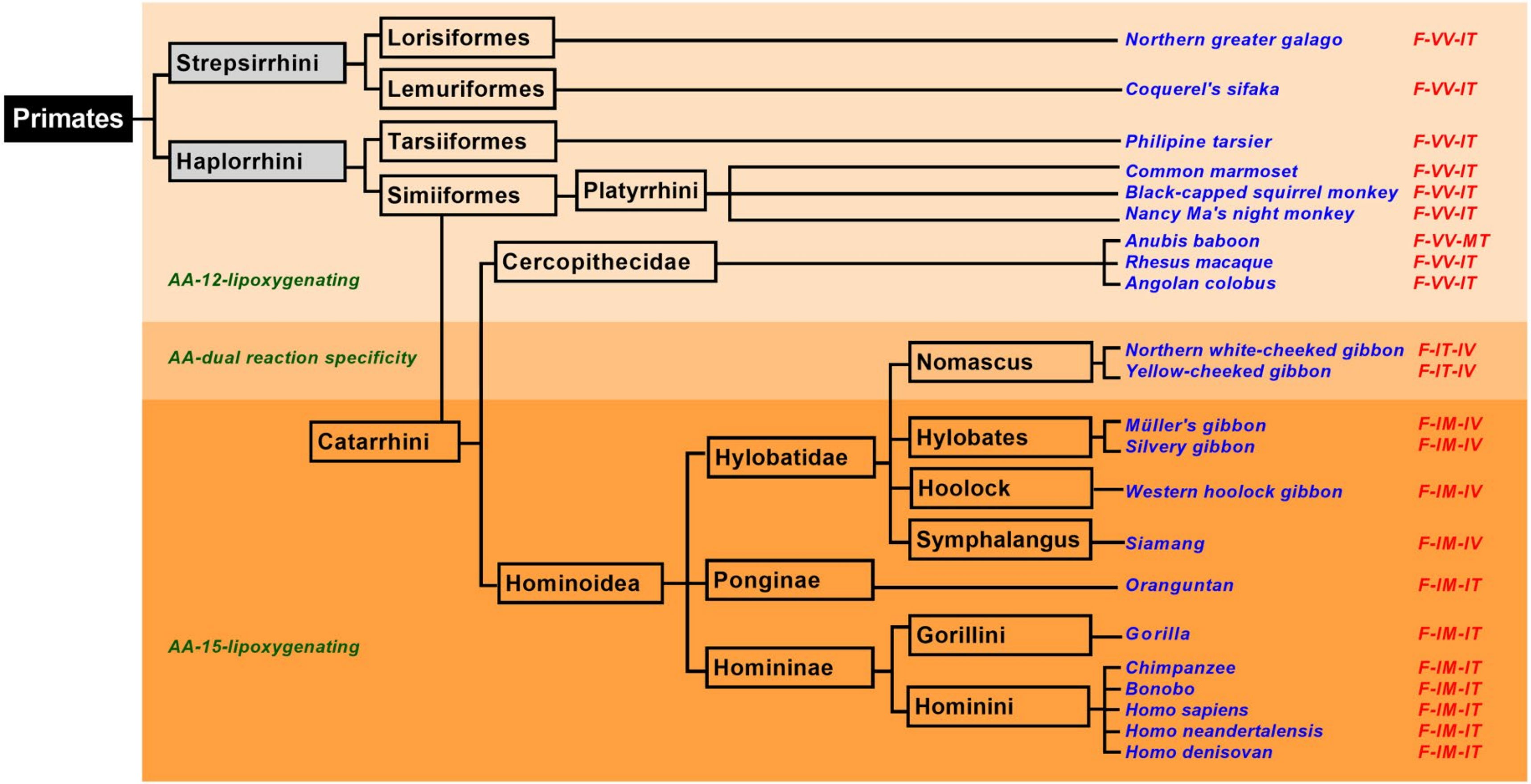

Movie S1: Impact of mutations of triad determinants on the structure and the deepness of the substrate binding pocket. This video clip is aimed at visualizing the gain of space that is achieved when the bulky triad determinants of rabbit ALOX15 are mutated to less space-filling residues present at these positions in AA 12-lipoxygenating ALOX15 orthologs. The additional space that is gained by these mutations can be used by arachidonic acid to penetrate deeper into the substrate binding pocket so that the substrate can adopt a position at the active site that favors C12-oxygenation. This video is based on the X-ray coordinates of the rabbit ALOX15 (PDB 1LOX) and is intended for visualization purpose only. We did not include MD simulation data on substrate binding at the active site of wildtype rabbit ALOX15 or any of the mutants shown in this video. Please start the video now by clicking the star button. The overall structure of the enzyme resembles a cylinder with a height about 10 nm and an elliptic ground square (long diameter 6.1 nm, short diameter 4.5 nm). It consists of two domains. The small N-terminal domain (light green) consists of several ß-sheets (not shown in this video). The large C-terminal domain (dark green) involves the substrate binding pocket with the catalytic non-heme iron. The entrance into the substrate-biding pocket is located at the surface of the C-terminal domain and Gly407 (G407), Leu597 (L597) and Arg403 (R403) are lining the entrance. Looking into the entrance whole one can actually see the catalytic non-heme iron, which is represented by the green sphere labeled with Fe. Arg403 (R403) carries a flexible side chain and has previously been suggested to interact ionically with the carboxylic group of the fatty acid substrate. However, more recent mutagenesis studies did not confirm this hypothesis. Substrate fatty acids slide into the substrate binding pocket of rabbit ALOX15 with their methyl end ahead (tail-first substrate orientation). For this video, we put a little camera on the CH_3-carbon of a polyenoic fatty acid and allow the substrate moving into its binding pocket. The path of the camera is indicated by the yellow trace. After passing the non-heme iron the camera turns towards the bottom of the U-shaped substrate binding pocket and then moves back to have a look at the triad determinants [Ile(I)593, Phe(F)353, Ile(I)418], which form the bottom of the U-shaped substrate binding pocket. Along this way, the camera first passes the catalytic non-heme iron, which is liganded by His361 (H361), His366 (H366), His541 (H541), His 545 (H545) and the N-terminal Ile663 (I663). The distances between the liganding atoms and the central iron are given in nm. It should be stressed, that the octahedral iron ligand sphere is completed by a water molecule or a hydroxy ligand, which occupies the sixth ligand position but is not shown in this presentation. After the camera has passed the iron ligand sphere it takes a turn moving towards the bottom of the substrate binding pocket. On its way it first approaches Ile593 (I593, the third triad determinant) and mutation of this amino acid to a less space-filling Ala opens additional space allowing the substrate fatty acid to penetrate deeper into the substrate binding pocket. Next, the camera meets Phe353 (F353). Here again, mutation of this bulky amino acid to less space-filling Ile(I) [Phe(F)353Ile(I)] provides additional space that can be used by the fatty acid substrates to move deeper into the substrate binding pocket. If Phe353 (F353) is mutated to an even less space-filling Ala(A) (not shown in the video), even more space is provided and the alterations in the product patterns are frequently more pronounced **(Table S2)**. Finally, the camera meets Ile418 (I418), the major Sloane determinant, and similar mutations at this residue [Ile(I)418Ala(A)] also provides additional space for deeper penetration of the substrate fatty acid.
